# Educational Strategies for Managing Moral Distress in Student Nurses: A Scoping Review

**DOI:** 10.1111/jan.70320

**Published:** 2025-11-03

**Authors:** Rebecca Timmins, Chris Kite

**Affiliations:** ^1^ School of Nursing Faculty of Education, Health and Wellbeing, University of Wolverhampton Wolverhampton UK; ^2^ School of Health and Wellbeing Faculty of Education, Health and Wellbeing, University of Wolverhampton Wolverhampton UK; ^3^ Division of Public Health, Sport and Wellbeing Faculty of Health, Medicine and Society, University of Chester Chester UK; ^4^ Warwickshire Institute for the Study of Diabetes, Endocrinology and Metabolism (WISDEM), University Hospitals Coventry and Warwickshire NHS Trust Coventry UK

**Keywords:** constraints, moral courage, moral decision making, moral distress, moral resilience, moral sensitivity, nurse education, student nurses

## Abstract

**Aims:**

To explore what content, teaching and learning activities are advocated by nurse educators to mitigate moral distress and related concepts in student nurses.

**Design:**

Scoping review.

**Review Methods:**

The review was conducted according to Joanna Briggs Institute guidelines. The search strategy adopted their three‐step method for systematic reviews. The eligibility criteria reflected the Population, Concept, Context format.

**Data Sources:**

CINAHL Ultimate, MEDLINE Full Text, APA PsycINFO, Education Research Complete, Web of Science, ProQuest, Base, and Open Grey were systematically searched in September 2024 for papers in English language regardless of publication age.

**Results:**

Following searches, 3809 records were screened against eligibility criteria, resulting in 42 eligible papers being included; 29 research studies and 13 non‐empirical papers. We identified 236 content suggestions, mapped to 70 subject codes. Also, 217 teaching and learning activities are suggested and mapped to 41 coded activities. Data is charted in tables and figures and results are discussed per related concept of moral distress.

**Conclusions:**

Educational content, and teaching and learning activities are heterogenous across the concepts influencing moral distress. There is overlap of content across different concepts. Moral sensitivity received the most publications. Development of research and educational strategies addressing other interrelated concepts would be advantageous for evidence‐based curriculum development. Recommendations are made to develop evidence‐based content and teaching and learning activities.

**Implications for the Profession and/or Patient Care:**

Recommendations are made to develop an evidence‐based multi‐conceptual curriculum to mitigate moral distress in pre‐registration student nurses.

**Impact:**

Recommendations are made adding to existing research agendas on the topic.

**Reporting Method:**

PRISMA‐ScR.

**Patient or Public Contribution:**

No patient or public contribution.

## 1 Introduction

The term moral distress was coined by Jameton (1984) following discussions with student nurses and defined as students knowing the right thing to do, but constraints and/or conflicts with co‐workers made it impossible for them to pursue the right course of action. The negative responses of moral distress in student nurses are evidenced internationally (Bickhoff et al. 2017; Sasso et al. 2016) and recognize a student's increased risk of suicide and depression (Paidipati et al. 2023), frustration, and guilt (Escolar Chua and Magpantay 2019; Sasso et al. 2016), with some so affected that they want to leave the profession (Yilmaz and Kiziltepe 2022). A negative impact on patient care is also reported due to students experiencing moral distress (Bickhoff et al. 2017; Yilmaz and Kiziltepe 2022).

In mitigating the adverse effects of moral distress in student nurses, the need for educational interventions is frequently cited (Heng and Shorey 2023; Rushton et al. 2017; Sasso et al. 2016). Whilst there has been progress in developing interventions to address moral distress, these are often multifaceted and take the form of a variety of bundles of interventions due to the diffuse nature of moral distress, and it is often unclear which intervention has what effect on moral distress (Morley et al. 2021).

This review aims to assist those involved in nurse education internationally, who serve a crucial role in the preparation of nurses and can use teaching strategies to address moral distress during student nurse training (Loyd et al. 2023). Employing a scoping review methodology to disentangle the proposed content and teaching and learning activities (TALA) embedded within multicomponent interventions aimed at mitigating moral distress serves as a foundational step toward developing a comprehensive, evidence‐based curriculum to address this pervasive experience among students. This review aims to identify the range of educational content, defined as a body of disciplinary knowledge (Biggs and Tang 2011), and TALA, defined as activities that facilitate students achieving the desired educational outcome (Biggs and Tang 2011), advocated in nurse education to address moral distress.

A concept analysis of student nurses' experiences of moral distress found other interrelated concepts manifested which influenced the student nurses' experience of moral distress (Timmins 2024). Some students displayed moral sensitivity, the ability to recognize a moral conflict and have insight into the ethical consequences of the moral conflict for the person (Lutzen et al. 1995); this is often influenced by ethics knowledge. A study by Goyaghaj et al. (2021) found moral sensitivity plays a role in predicting moral distress in nurses. Escolar Chua (2018) found that when student nurses are morally sensitive, the greater their predisposition to act as moral agents. Therefore, promoting the moral sensitivity of nursing students can increase their moral competence and contribute to decreasing the impact of moral distress.

Studies have also shown that moral sensitivity is positively correlated with ethical decision‐making and mediated by strong professional values (Chen et al. 2021). Nursing students are vulnerable to moral distress when faced with ethical dilemmas or decision‐making in clinical practice (Sasso et al. 2016). Improving students' ethical decision‐making skills, therefore, can influence their moral competence and experience of moral distress. Conceptually, Jameton's (1984) definition of moral distress requires knowing the right thing to do, requiring cognitive skills that facilitate making judgments or analyzing ethical conflicts prior to decision making (Torabizadeh et al. 2016). However, even if nursing students master the skills of recognizing ethical conflicts (moral sensitivity), develop competence in moral judgment and decision making to do the right thing, it does not mean that they will act in the right way. The literature demonstrates that whilst students may know the right thing to do, at times they may act as passive observers of poor care (Bickhoff et al. 2017) and are thus not exhibiting moral courage, another concept influencing the students' experiences of moral distress.

Moral courage, in contrast to doing nothing, is the willingness to take a stand that challenges the health care organisation or those in it, even when a person's job may be jeopardised (Fry 1989). Moral sensitivity and decision making are prerequisites to moral courage (Koskinen et al. 2021), and exhibiting moral courage can reduce moral distress (Numminen et al. 2017). A study by Escolar Chua (2018) found students' moral distress intensity was positively correlated to multiple subscales of the moral courage scale, concluding that a student's willingness to exhibit moral courage can depend on the moral intensity of the situation. Arguably, students should exhibit moral courage in all morally reprehensible situations and not just those that provoke such feelings of intensity.

Barriers to taking the right course of action and displaying moral courage have been found to be due to internal and external constraints (Epstein and Hamric 2009). Jameton (1993) gives examples of external constraints such as poor communication between team members, pressure to reduce costs, fear of legal action, lack of administrative support, and hospital policies that conflict with patient care needs. Internal constraints are suggested to occur within the individual, and in students were found to involve avoiding conflict to preserve student learning (Bickhoff et al. 2017) and to preserve the student's relationship with their supervisor (Krautsheid et al. 2020).

In considering educational interventions to address moral distress, it would be prudent to consider a comprehensive approach addressing the interrelated concepts that potentiate moral distress such as moral sensitivity, decision making, and courage in mitigating moral distress, rather than managing the symptoms of moral distress. Therefore, and in line with the aims of scoping reviews (Munn et al. 2022), this review was performed to identify the types, extent, and variety of available evidence in the published and grey literature, and to describe the key characteristics of educational content and TALA proposed by nurse educators to address moral distress and its influencing concepts in the student nurse population.

## 2 Methods

In line with JBI guidelines (Peters et al. 2020), a scoping review protocol was developed in Spring 2024 under the supervision of the first reviewer's supervisory team and prior to conducting the review (Data S1). This review is also reported according to the Preferred Reporting Items for Systematic Reviews and Meta‐Analyses: extension for Scoping Reviews (PRISMA‐ScR) checklist (Tricco et al. 2018) (Data S2).

### 2.1 Research Questions

The two research questions guiding this review were developed to incorporate the Population, Concept, Context (PCC) framework as recommended in the JBI Standards (Peters et al. 2015). The questions are:

1. What educational content is proposed and/or implemented by nurse educators (context) to mitigate the negative impact of moral distress and influencing concepts (concept) in student nurses (population)?
2. What teaching and learning activities are proposed and/or implemented by nurse educators (context) to mitigate the negative impact of moral distress and influencing concepts (concept) in student nurses (population)?

### 2.2 Eligibility Criteria

Eligibility criteria were devised using the PCC Framework (Peters et al. 2015) (see Protocol Table S1 for detailed criteria). The review included all papers where educational interventions were proposed/implemented by Nurse Educators to mitigate moral distress or concepts influencing moral distress. Papers published in languages other than English were excluded due to resource constraints, but no time range limits were applied. Sources within peer‐reviewed journal articles and theses (i.e., Grey Literature) were included (See ScR Protocol Table S1).

### 2.3 Search Strategy

The search strategy adopted was conducted in consultation with a health librarian at the University. The search strategy took the form of the three‐step method (Peters et al. 2020) and was conducted in September 2024. The initial search was conducted in two databases (CINAHL Ultimate and MEDLINE) to analyze the title and abstract of retrieved papers and of the index terms used to describe the articles. A search strategy was then developed from the initial search with key terms and index terms, and a second search was performed across four databases: CINAHL Ultimate, MEDLINE, PsycInfo, and Education Research Complete (see Table 1, full search strategy, Table 2, PCC Search strategy). The reference list of all full‐text papers sought and papers included in the review were searched for additional sources and eligible papers.

**TABLE 1:** Search strategy Scoping Review: Educational Strategies for Managing Moral Distress in Student Nurses: A Scoping Review.

Search Number	Search Terms
#1 #2 #3 #4 #5 #6 #7 #8 #9 #10 #11 #12 #13 #14 #15 #16 #17 #18 #19 #20 #21 #22 #23 #24 #25 #26 #27 #28 #29 #30 #31	Nurs* Student* (Title/Abstract) Bachelorette (Title/Abstract) Undergraduate nurs* (Title/Abstract) Students, Nursing [MeSH] #1OR #2 OR #3 OR #4 Intervention (Title/Abstract) Education (Title/Abstract) Address (Title/Abstract) Alleviate (Title/Abstract) Reduce (Title/Abstract) Mitigate (Title/Abstract) Nurse Educat* (Title/Abstract) Teach* (Title/Abstract) Tutor (Title/Abstract) Education Nursing [MeSH] Nursing Faculty [MeSH] Nurses [MeSH] #6 OR #7 OR #8 OR #9 OR #10 OR #11 OR #12 OR #13 OR #14 OR #15 OR #16 OR #17 “Moral Distress” (Title/Abstract) “Moral Stress” (Title/Abstract) “Ethics stress” (Title/Abstract) “Ethical Distress” (Title/Abstract) “Moral Sensitivity” (Title/Abstract) “Ethical Sensitivity” (Title/Abstract) “Moral Courage” (Title/Abstract) “Poor patient care” (Title/Abstract) “Harm to patient” (Title/Abstract) “Unsafe care” (Title/Abstract) “Internal Constraints” (Title/Abstract) “External Constraints” (Title/Abstract) #19 OR #20 OR #21 OR # 22 OR #23 OR #24 OR #25 OR #26 OR #27 OR #28 OR #29 OR #30
#32	#5 AND #18 AND #31

**TABLE 2:** Population, context, concept, search strategy.

Construct	Search terms
Population	Nurs* Student* OR Bachelorette OR Undergraduate nurs* OR Students, Nursing [MeSH]
	AND
Context	Intervention OR Education OR Address OR Alleviate OR Reduce OR Mitigate OR Nurse Educat* OR Teach* OR Tutor OR Education Nursing [MeSH] OR Nursing Faculty [MeSH] OR Nurses [MeSH]
	AND
Concept	Moral Distress OR Moral Stress OR Ethics stress OR Ethical Distress OR Moral Sensitivity OR Ethical Sensitivity OR Moral Courage OR Poor patient care OR Harm to patient OR Unsafe care OR Internal Constraints OR External Constraints

Grey literature was searched via Web of Science, ProQuest, Base, and Open Grey. The search string was simplified in these databases and included the terms “Moral Distress” and “Education”. Literature alerts were set up from September 2024 to notify of any emerging literature on the topic.

### 2.4 Screening and Study Selection

Search results were exported from databases into review manager software Rayyan (Ouzzani et al. 2016), and duplicates were removed. Title and abstract screening were completed independently by two reviewers, and ineligible papers were excluded. The same two reviewers independently screened the full‐text articles, and where there were disagreements, they were resolved through discussion; arbitration from another reviewer was not required as consensus was reached for all disputed papers.

### 2.5 Extracting Data

A standardised data extraction form was created to capture data relevant to the review's research questions. Extracted fields included author, date of publication, country of origin, aims/purpose, population, sample size, sampling method, study design, intervention type, concept(s) addressed, educational content, TALA, outcomes, key findings, and limitations (Table 3, Summary table of papers). In line with the aim of scoping reviews to map and summarise the available evidence, frequency counts, percentages, and basic coding were performed. First cycle descriptive coding, as described by Saldana (2009), involving summarising in a word the basic topic of educational content and TALA, which is aligned to the ScR objectives and PCC, aided organising qualitative data into categories. One reviewer (RT) immersed themselves in the data, familiarising themselves, iteratively assigning and reassigning codes, and performing intra‐rater reliability activities (i.e., use of a code book, decisions for coding, recoding). Once complete, all codes and the corresponding themes were verified through discussion with the second reviewer (see Data S3, Coding Table of Content and TALA). Once extracted, data were presented using tables and figures, and a narrative synthesis was also presented.

**TABLE 3:** Summary table of papers.

Author, date and title	Key information	Related concept of MD addressed in title/abstract	Content and content basic codes in bold	TALA and TALA basic codes in bold
Azarkish et al. (2023) *Comparison of the effect of teaching professional ethics codes using the two methods of the flipped classroom and short message service on the moral sensitivity of nursing students*	**Country**: Iran **Setting**: University **Participants**: 120 nursing students (convenience sampling) **Study design**: A quasi‐experimental design	Moral sensitivity	**Professional and Ethical codes**. Content same for all groups but method of delivery different	Interventions 1 (**flipped classroom/PBL**, recorded **lectures** for each session, accompanied by **Slides and** images, instructional **videos**, and **concept maps** with 1st‐semester students) Intervention 2 (Short messaging service/WhatsApp service, links to **instructional videos**, and **reading articles to 5th semester students**) Control group (3rd‐semester students) **ethics codes** education
Baykara et al. (2015) *The effect of ethics training on students recognising ethical violations and developing moral sensitivity*	**Country**: Turkey **Setting**: University **Participants**: 50 nursing students **Study design**: Randomised Control Trial (RCT)	Moral sensitivity	Control group and experimental group both took Nursing Practice Course‐II (content not discussed). Experimental group received extra training on **ethical codes**, ethical responsibilities, ethical violations, and the precautions to prevent them **Professional and Ethical codes** **Ethical principles, patient rights, drug administration errors. Ethics in nursing practice. Legal issues, research ethics**	**Question and answer methods, and case studies** were used to provide extra training to the experimental group Multi modal interventions to explore impact on moral sensitivity
Ciesielski (2022) *Nurse Educator Perspectives on Undergraduate Nursing Curriculum and Moral Courage Development*	**Country**: US **Setting**: University **Participants**: Eight nurse educators. **Study design** Qualitative descriptive study (Grey Lit) US	Moral courage and Moral distress	**Scarce content** on moral courage and moral distress was found to exist in the nursing curriculum which the eight educators taught upon **Ethical decision‐making skills. Ethics in nursing practice**	Nurse educators **spoke to students (discussion)** after taught sessions about their experiences of moral distress. Nurse educators **taught students about Hidden curriculum activities** Nurse educators proposed **reflection, and reflective journaling** to aid discussion about moral distress Should be taught **didactically** and in clinical practice **Role modelling** moral courage
Ekramifar et al. (2018) *The Effect of Spiritual Training on the Moral Sensitivity of Nursing Students*	**Country**: Iran **Setting**: University **Participants**: 70 nursing students of the fourth and higher semesters **Study design**: Quasi‐experimental study	Moral sensitivity	**Spirituality, religion and faith training** Interventional group received training on the definition and differences between the concepts of spirituality, religion and faith. Characteristics of a spiritual person. Spirituality in nursing clinical practice. Relationship between spirituality and mental health. Religious‐spiritual confrontations Control group training not discussed	The subjects were taught through **lecturers, PowerPoint slides**, and **question and answer** by the researcher under the supervision of a supervisor. **Reading material and discussion**
Ertugrul et al. (2022) *The effects of an ethics laboratory program on moral sensitivity and professional values in nursing students: A randomised controlled study*	**Country**: Turkey **Setting**: University **Participants:** 100 nursing students (second grade) were assigned **Study design**: RCT	Moral sensitivity	The ethics laboratory developed ethical scenarios integrated with the subjects included in the fundamentals of nursing course 7 topics included: **Patient safety in nursing care:** Vital sign incompetence Unsafe discharge Unsafe pre procedure checks Unsafe urinary catheterisation procedure **End of life care** **Drug administration/error** **Nursing ethics codes** **Nursing ethics** Professionalism (added to Ethical and professional codes) **Informed consent/refusal** **Ethical decision making**	The 8‐week ethics laboratory programme was applied to the students in the intervention group (interactive education methods, such as ethical **scenarios**, **case studies, roleplay, group discussions, project papers and watching movies**). The control group received standard curriculum Control group received the standard fundamentals of nursing curriculum of nursing course were first taught via theoretical presentation (method not stated) and then, psychomotor skills were introduced through the professional skill laboratories (activity not stated) Multi modal interventions to explore impact on moral sensitivity
Ford et al. (2024) *Brave spaces in nursing ethics education: Courage through pedagogy*	**Country**: Canada **Setting**: Nursing students enrolled in a nursing healthcare ethics & law course **Participants**: 39 undergraduate nursing students. Senior year students. (Purposive sampling) **Study design**: Exploratory, cross‐sectional survey design	Moral courage	**Ethical and legal considerations in medical assistance in end of life, and beginning of life**, **conscientious objection**, p**andemic ethics, unethical practice**, **anti‐racism/discrimination, and implicit bias**	Brave spaces and the use of the self‐assessment tool ESA “Engagement Self‐Assessment” on the creation of **brave spaces and ground rules** in nursing ethics course **Self‐reflection** Activities beyond the ESA discussed
Forte et al. (2024) *Fostering Moral Resilience, Evaluating a High‐Fidelity Ethics Simulation with Prelicensure Nursing Students in Their Practice as New Graduates*	**Country**: US **Setting**: University **Participants**: 123 nursing students (Senior semester) participants **Study design**: Mixed methods. Exploratory design	Moral resilience is the focus	Didactic training in classroom: **Ethical principles (autonomy, beneficence, nonmaleficence, justice, and veracity**), and the concepts of **moral distress and moral resilience** Simulation involved reviewin**g ethical principles, and codes of ethics**. **Using naming, framing, and claiming framework** and using **sensory awareness skills** **Consent**	All students received **didactic** training and scenarios in the classroom about ethical principles Students in the experimental cohort also participated in a 2‐h **high fidelity ethics simulation**
Garity (2009) *Fostering nursing students' use of ethical theory and decision‐making models: teaching strategies*	**Country**: US **Setting**: Described ethics content in university **Participants**: N/A **Study design**: Not primary research	Moral distress	**Professional and Ethical Codes** **Ethical theories** (Aristotle, Socrates, Altruism, Duty‐Based, Rights‐Based, Virtue‐Based, and Utilitarianism) **Ethics and law at beginning of life** **Ethics and law at end of life** Pro‐choice Vs. pro‐life. Maintenance vs. withdrawal of treatment Quantity of life vs. quality of life. Euthanasia vs. non‐euthanasia **Ethical decision‐making models**	Teaching strategies include discussion questions, **case studies, textbook readings, PowerPoint slides, videos, assignment writing, debates**. **Discussion** **Journal clubs are proposed**
Ghoozlu et al. (2023) *Ethics education: Nurse educators' main concern and their teaching strategies*	**Country**: Iran **Setting**: Teachers of ethics in a university **Participants**: 11 nurse educators (Purposive sampling) **Study design**: Qualitative descriptive study	Moral sensitivity	**Ethical theory/principles** **Ethics in nursing practice**	Nurse educators use active learning techniques to teach ethics. They used **moral cases/case studies** to increase moral sensitivity To sensitise students to ethical nursing care, nurse educators try to institutionalise ethical principles using different teaching methods, through **simulated situations, role play, film** Multi modal interventions to explore impact on moral sensitivity
Guzys (2021) *Moral distress: A theorised model of influences to facilitate mitigation and resilience*	**Country**: Australia **Setting**: Not stated **Participants**: N/A **Study design:** Not primary research	Moral distress	**Code of ethics** **Ethical principles content** **Advocacy** **Critical thinking skills** **Nursing ethics** **Nursing philosophy** **Leadership**	**Reflection**
Harvey et al. (2021) *Using Tag Team Simulation for Ethics Education in Undergraduate Nursing Students*	**Country**: Canada **Setting:** University **Participants:** 2nd year nursing students. Number not stated **Study design**: Not primary research	Moral distress	**Informed consent and** impaired capacity **Ethical decision‐making framework** **Self‐care strategies (not specified)**	**Lecture** Tag Team **simulation** in ethics (including **debrief** and group feedback) **Decision making**
Howarth (2022) *Nurse Educators Teaching Nursing Ethics to Prepare Students for Moral Distress: A Descriptive Study*	**Country**: US **Setting:** Nurse educators teaching on nursing programmes in college or university **Participants**: 11 nurse educators (purposive sampling) **Study design**: Qualitative descriptive study. (Grey lit)	Moral distress	**Scarce content in curriculum** Recommendations include making **moral distress** content part of the formal nursing curriculum and teaching this content	**Case‐study** Signposting support **Discussing** moral distress with students
Jasemi et al. (2022) *Educating ethics codes by lecture or role‐play; which one improves nursing students' ethical sensitivity and ethical performance more? A quasi‐experimental study*	**Country**: Iran **Setting**: University **Participants**: 114 nursing students. Sixth, seventh and eighth semester students. **Study design**: Quasi‐experimental study	Moral sensitivity	Content was similar for both intervention groups and included (a) **ethical codes** of ethics in Iran, (b) definition of ethics, (c) the need for nursing students to be aware of **nursing ethics**, and (d) the importance of **ethics in nursing**	Delivery of content different. 1 intervention group (**role‐play**) 1 interventional group (**lecture**) and 1 control group (no intervention, only usual curriculum but curriculum was not described). 38 students in each group. **Scenarios**. **Question and answer**
Johansen et al. (2023) *Addressing moral injury in nursing education*	**Country**: US **Setting**: Not stated. **Participants**: N/A **Study design**: Not primary research	Moral injury	Recognition and support for MI: Content to enable ethical competence **Decision making** **Conflict management** **Ethics education** **4 A's rise to moral distress (ANA)** Causes of moral injury: **natural disasters**, climate change and others **Health and wellbeing**	**Lectures**. **Case Studies** **Discussions** **Safe environment. (Learning environment)** **Simulation** **Debriefing** **Lecturing** **Role modelling** **Ethics webinar** **Self‐care assignment** **Ethical competence**
Jones‐Schenk and Trepanier (2021) *R U OK?*	**Country**: US **Setting**: Intervention performed in the author's College of Health Professions **Participants**: N/A **Study design:** Nor primary research	Moral distress	R U OK? Campaign (**Health and wellbeing**) Recognise and address **moral distress toolkit** (AACN 2020)	**Discussion** led by faculty staff directed to students
Khatiban et al. (2019) *Lecture‐based* versus *problem‐based learning in ethics education among nursing students*	**Country**: Iran **Setting**: University **Participants**: 66 nursing students from seventh and eighth semester **Study design**: Quasi‐experimental study	Moral decision making	**Codes of nursing ethics** **Patient rights** **Decision‐making** in solving ethical problems, and six **scenarios** of moral dilemmas but these were situated in research tool used and not intervention (NDT)	Lecture V Problem Based Learning (PBL) **Lecture** and **PowerPoint presentation** **Problem based learning** Ethical **scenarios** **Reading material**
Kim and Park (2019) *The effects of debate‐based ethics education on the moral sensitivity and judgement of nursing students: A quasi‐experimental study*	**Country**: South Korea **Setting**: Students attending two universities with similar syllabi were recruited into study **Participants**: 64 nursing students. Senior year **Study design**: Quasi‐experimental study	Moral sensitivity and moral judgement	Ten dilemma cases including **Ethics at beginning of life** **Ethics at end of life** **Ethics in mental health nursing Confidentiality** **Interpersonal relationships (constraints)** **Conflict between hospital policies and nurses' views (constraints)** **Ethical theories: Utilitarianism, deontology, autonomy, non‐maleficence, beneficence, justice, honesty, fidelity, confidentiality, and privacy** **Ethical decision‐making** **Codes of ethics for nurses**	ADDIE **model Debate**‐based ethics education versus **lecture** style education
Kucukkelepce et al. (2020) *Effects of using standardised patients on nursing students' moral skills*	**Country:** Turkey **Setting**: University **Participants:** 89 nursing students (convenience sample) **Study design**: Quasi experimental study	Moral sensitivity, moral reasoning judgement	Theoretical teaching: **ethical concepts, principles**, **theories, nursing ethics**, human and **patient rights. Bioethics: Newborns right to life** Five cases were used as scenarios but from not intervention in NDT Tool	Both attended ethical theory **lecture**. Group 1 learning with standardised patients (**simulation):** Group 2 were taught using the **case study** analysis method in the classroom Multi modal interventions to explore impact on moral sensitivity
Lee and Huang (2017) *Evaluating the effect of three teaching strategies on student nurses' moral sensitivity*	**Country**: Taiwan **Setting**: Year 3 students of a 5 year college nursing programme **Participants**: 234 nursing students **Study design**: Quasi‐experimental study	Moral sensitivity	**Nursing ethics** Reading **Nightingale's Diary**. Impact of kidney **transplant** patients' life. **Ethical decision making (Four topic approach)**	VAK model (Visual, Auditory, Kinaesthetic) approaches to teaching and learning activities on ethics Visual—**Reading** textbook. In person **discussion**. Clinical **scenarios** Kinaesthetic – writing **assignment**. Touching patient's AV shunt. **Lit searching**. Group **project** Auditory—Patient dialogue regarding transplantation/**patient experience**. Group **discussion**. **Brainstorming** **Case study** Multi modal interventions to explore impact on moral sensitivity
Maddineshat et al. (2018) *Teaching ethics using games: Impact on Iranian nursing students' moral sensitivity*	**Country:** Iran **Setting**: University **Participants**: 30 nursing students. Second semester **Study design**: Quasi‐experimental study	Moral sensitivity	Professional ethics education **Bioethics** **Nursing Ethics** **Communication models** **Ethical decision making** **Legal issues** **Codes of Ethics**	**PowerPoint slides** **Mobile phone technology** **Game playing** of ethical scenarios to enable learning (e.g., brainstorming, buzz group, card sorting) **Lecture** **Assignment** **Debates** **Case Study** **Field visits** Multi modal interventions to explore impact on moral sensitivity
Mattsson (2024) *Challenges in integrating ethical theory into gerontological nursing care during students' first clinical rotation: A discussion paper*	**Country**: Sweden **Setting**: Nurse educators' reflections on nursing students' assignment narrative content **Participants**: 1st year nursing students **Study design**: Not primary research	Moral courage	**Codes of Ethics** **Ethical theories (not specified)** **Decision making** **Practical nursing care skills** **Ethics in death and dying/end of life** **Palliative care** **Moral development** **Gerontology ethics**	**Assignments**. **Online discussion**. **Reflection** Student centred approaches proposed – not specified
Monteverde (2016) *Caring for tomorrow's workforce: Moral resilience and healthcare ethics education*	**Country**: Switzerland **Setting**: Educational setting **Participants**: 166 nursing students (purposive sampling) **Study design**: Quantitative nonrandomised pre/post interventional study	Moral resilience	Morally wrong or morally complex situations **Ethical theories** **Ethical reasoning/decision making** Compromised care due to understaffing(External **constraints**) **Drug admin/errors** **Ethical issues in mental health nursing** (Involuntary restraint/issues in dementia care)	**Vignettes (Case teaching)** **Lecture**
Morrill and Westrick (2022) *Fostering Nursing Students' Moral Decision‐Making Through Use of an Affective Learning Module*	**Country**: US **Setting**: Describes module used for nursing students on BSc programme **Participants**: N/A **Study design**: Not primary research	Moral sensitivity and decision making	Rest and Narvaez (1994) **4 component model to structure content** (Integrity, core nursing values, ANA code of Ethics, Standards of Practice) **Professional codes of ethics**	**Lecture** **PowerPoint/slides** **Reflection/reflective writing** **Case study** **Discussion**. **Role modelling**
Nesime and Belgin (2022) *Impact of Education on Student Nurses' Advocacy and Ethical Sensitivity*	**Country:** Turkey **Setting**: University **Participants:** 80 senior fourth grade third year undergraduate nursing students **Study design**: RCT. (CONSORT criteria reporting and protocol registered)	Moral sensitivity	Routine education curriculum (content not stated) V advocacy education **Advocacy education** and theoretical education (i.e., social justice, **inequality/social determinants in health**) **Communication and counselling skills**	**Case study** **Action plan** **Poster presentation** **Slide presentation** **Movies/Videos/visual material** **Assignment** **Quiz** **Question/answer** **Exam** **Observation** Multi modal interventions to explore impact on moral sensitivity
Park (2011) *The relationship of ethics education to moral sensitivity and moral reasoning skills in Baccalaureate Nursing Students of South Korea*	**Country**: South Korea **Setting:** Eight private Bachelorette programmes in South Korea **Participants**: 946 nursing students (506 freshman, 440 senior students). Purposively sampled **Study design**: Quantitative nonrandomised study. (Grey lit)	Moral sensitivity and moral reasoning	**Ethics contents with clinical practice** **Ethical frameworks** (Duty and Virtue ethics) **Codes of Ethics** **Nursing Ethics** **Ethical decision making** **Ethics in nursing practice**	**Lectures** **Group discussion** **Case analysis (Case teaching)** **Role play** **Problem‐based learning** Multi modal interventions to explore impact on moral sensitivity
Park et al. (2012) *The relationship of ethics education to moral sensitivity and moral reasoning skills of nursing students*	**Country:** South Korea **Setting**: Eight private Bachelorette programmes in South Korea **Participants**: Participants: 946 nursing students (506 freshman, 440 senior students). Purposively sampled **Study design**: Quantitative nonrandomised study	Moral Sensitivity and moral reasoning	Ethics content as a separate course or integrated into existing curriculum	**Lecture** Group **discussions**
Parker and Welch (2024) *RAISE Your Graduate to Support a Healthy Work Environment, Teaching and Learning in Nursing*	**Country**: US **Setting**: Nurse educators discuss strategies to reduce moral distress **Participants**: N/A **Study design**: Not primary research	Moral distress	**ANA Healthy work environment** **Professional and ethical codes** **AACN 4 A's Model** **Moral Distress Thermometer** **University wellbeing support services** **RAISE programme**	**Ethics consultation** **Nurse educator mentors/coaching** **Role modelling** **Reflection**
Parsh (2021) *What is Moral Distress?*	**Country**: US **Setting:** Discusses student case study experiencing MD **Participants**: N/A **Study design**: Not primary research	Moral Distress.	**4 A's rise to moral distress** **Ethics committees** **Wellbeing:** work‐life balance **Self care:** Code lavender Mindfulness based stress reduction	**Debriefs**
Qu et al. (2024) *The effect of simulated problem learning in nursing ethics on moral sensitivity, empathy and critical thinking of nursing students: A quasi‐experimental study*	**Country:** China **Setting**: University **Participants:** 161 nursing students. **Study design**: Quantitative nonrandomised study	Moral Sensitivity	Ethics courses content: **Interpersonal relationships** **Ethics: In nursing practice** **Public health services, In medical technology** **In end of life** **In nursing research** **In nursing management**	**Simulation with problem‐based learning** Learning (Intervention) V **Problem‐based learning** (Control group) **Lecture** **Videos** **Case presentations/case study** **Slides** Multi modal interventions to explore impact on moral sensitivity
Robichaux et al. (2022) *Ethics Education for Nurses: Foundations for an Integrated Curriculum*	**Country**: US **Setting:** Framework is discussed to address ethics education across levels of curricula and practice **Participants**: N/A **Study design**: Not primary research	Moral Distress	**Professional and ethical codes** **Ethical decision‐making frameworks** **Ethics consultation/committee** **Professionals identify formation** **Emotional intelligence** **Self‐efficacy** **Advocacy skills** **Conflict resolution**	**Reflection** **Didactic activities (not specified)** **Debriefing** **Simulation** **Role play** **Discussion** **Role models** **Mentoring**
Rushton et al. (2017) *Executive Summary: Transforming Moral Distress into Moral Resilience in Nursing*	**Country**: US **Setting**: Reporting educational recommendations from symposium. **Participants**: N/A **Study design**: Not primary research	Moral distress, moral resilience	**Standardised basic ethics content** **Moral resilience content** **Ethical frameworks/theories**	**Ethics competencies (students and faculty)**
Sedgwick et al. (2019) *Analysis of Undergraduate Nursing Students' Sensitivity to Micro ethical Dilemmas During Simulation*	**Country**: Canada **Setting**: University simulated environment **Participants:** 68 third‐ and fourth‐year undergraduate nursing students **Study design**: Mixed‐methods convergent parallel design	Moral sensitivity	Ethical dilemmas (dilemmas not reported) arose whist simulating **routine nursing care/nursing practice** for patients with medical health conditions (i.e., diabetic ketoacidosis, sepsis, and congestive heart failure) **Ethics in nursing practice**	**Simulation** **Debriefing**
Torabizadeh et al. (2016) *Impacts of Socratic questioning on moral reasoning of nursing students*	**Country**: Iran **Setting**: University **Participants**: 103 nursing students. **Study design**: Quantitative nonrandomised study	Moral reasoning	**Ethics in nursing practice** **Ethical theories (**non‐explicitly stated)	**Lecture** **Scenarios** **Socratic questioning** (group sessions) (experimental1), or workshop method (experimental 2), or no intervention (control group) **Reflection**
Townsend and Cummings (2020) *Incorporating moral resilience into an undergraduate nursing program*	**Country:** US **Setting**: University nursing student programme **Participants**: 142 nursing students. Second semester, third and fourth semester students. Convenience sample **Study design**: Grounded Theory	Moral resilience	**Mental health first aid** **Moral distress content** **Moral resilience** **Compassion fatigue** **Incivility (constraints)** **End of life ethical issues** **Beginning of life ethical issues** **Wellbeing (mindfulness, meditation, yoga, healthy living)**. **Healthy work environment**	**Videos/visual material** **Scenarios** **Assignment** **PowerPoints** **Discussion** **Case Study** **Reflection** **Lecture** **Role play** **Simulation** **Reading material**
Uncu and Gunes (2021) *The importance of moral sensitivity in nursing education: A comparative study*	**Country**: Turkey **Setting:** University **Participants:** 461 nursing students **Study design:** Quantitative nonrandomised	Moral sensitivity	**Ethical theories/frameworks (Deontology ethics**) Ethical dilemmas (not specified)	**Discussion** **Case analysis/case study** Multi modal interventions to explore impact on moral sensitivity
Wawersik et al. (2023) *Perspectives on developing moral courage in pre‐licensure education: A qualitative study*	**Country**: US **Setting**: Interviews of Health care professionals to inform pre‐registration nursing education **Participants**: 14 health care professional educators **Study design**: Qualitative descriptive	Moral courage	**Errors and reporting/Human factors** **Ethical and legal implications** **TeamSSTEPS**	**Role modelling** **Learning environment (i.e., Ground rules, safer environment and psychological safety)** **Case studies** **Reflection** **Exam questions** **Debriefing** **Discussions** **Role play** **Simulation** **Coaching**
Wros et al. (2021) *Moral distress in public health practice: Case studies from nursing education*	**Country**: US **Setting**: Community public heath clinical placement **Participants**: 1055 nursing students **Study design:** Not primary research	Moral resilience, moral distress	**Wellbeing (i.e., Managing moral distress, mindfulness)** **Moving from Moral Distress to Moral Courage programme** **Ethics education (Bioethics, organisational ethics and micro ethics)** **Interprofessional collaboration** **Ethical discourse** **Moral climate** **Personal and professional values** **I‐Can programme** **Social determinants of health (external constraints)** **End of life case studies** **Bioethics (Justice in access to organ transplant)** **Professional codes of ethics** **Advocacy**	**Case studies** **Debriefing** **Role modelling** **Creation of safe spaces (learning environment)** **Reflection** **Mentorship** **Coaching** **Moral community development**
Yeom et al. (2017) *Effects of ethics education on moral sensitivity of nursing students*	**Country:** South Korea **Setting:** University **Participants**: 70 nursing students. Convenience sample **Study design:** Quantitative nonrandomised study. Quasi‐experimental design	Moral sensitivity	**Theories on ethics** & Principles. Rules of **nursing ethics** **Critical thinking** **Ethical dilemmas in nursing practice** **Ethical decision making** **Professional and ethical code of nursing** **Telehealth ethics** **Bioethics (Ethics at beginning of life, Abortion ethics, Reproductive technology ethics, Genetic screening)** **Children's issues:** Child abuse Protection of privacy for Minors **Refusal of treatment** **Organ transplant** **Ethics at end of life:** Euthanasia Withdrawal of treatment **Ageism (discrimination)** **Stigma in mental health nursing** **Chronically ill patient nursing issues**	**Lecture** **Group discussion** **Case analysis/case study** **Watch film/visual material** Multi modal interventions to explore impact on moral sensitivity
Yoes (2012) *Addressing Moral Distress, Challenge and Strategies for Nursing Faculty*	**Country:** US **Setting**: Faculty at College for Nursing **Participants:** N/A **Study design**: Not primary research	Moral Distress	**Conflict (care fronting)** **4 A's rise to moral distress model** **Self‐care (Health and wellbeing)**	
Yuksel Kacan (2022) *The Effect of Transcultural Nursing Course on Students' Moral Sensitivity: A Quasi‐Experimental Study*	**Country:** Turkey **Setting:** University **Participants**: 100 nursing students. Second Year **Study design**: Quantitative nonrandomised study. Quasi‐experimental design	Moral sensitivity	**Transcultural Nursing Course (TNC)** Content including: Health and culture Nursing and culture History and development of transcultural nursing Communication and cultural sensitivity Religion **Ethical Codes** **Ethics at beginning of life:** Cultural approaches to reproductive health, prenatal and postnatal period, infertility Childcare**/Children's issues** Chronic diseases**/Chronically ill** **Dilemmas in mental health nursing**: Psychiatric diseases Interventional group received TNC, control group did not receive TNC	**Role playing** **Case analysis/case study** **Video watching/visual material** **Article reading/reading material** **Discussion** Multi modal interventions to explore impact on moral sensitivity
Zia et al. (2023) *Problem‐based learning* versus *reflective practice on nursing students' moral sensitivity*	**Country**: Iran **Setting:** University **Participants:** 74 nursing students. 3rd and 4th year **Study design**: RCT	Moral sensitivity	**Professional and ethical codes** **Patient rights** **Ethical decision making** **Communication**	**PowerPoint slides** **Problem based learning (PBL)** **Reflection** **Scenarios** Study evaluates professional ethics training via methods of PBL, reflection or control group (given no instruction)
Ziyai et al. (2024) *The effect of flipped‐jigsaw learning models on ethical decision‐making*	**Country**: Turkey **Setting**: University **Participants:** 128 nursing students. 2nd year students **Study design**: Mixed methods nested design. Quasi‐experimental design, and a case study	Moral sensitivity and Moral decision making	**Ethical decision making** Ethical dilemma's relating to: **Beginning of life**, **Drug administration**, **End of life**, **Veracity**, **New workforce**	**Flipped classroom** **Jigsaw learning models** Ethics **reading material** resources (i.e., articles, books, journals) **Videos/visual material** **Case studies** Multi modal interventions to explore impact on moral sensitivity

## 3 Results

### 3.1 Search Results

**FIGURE 1:**
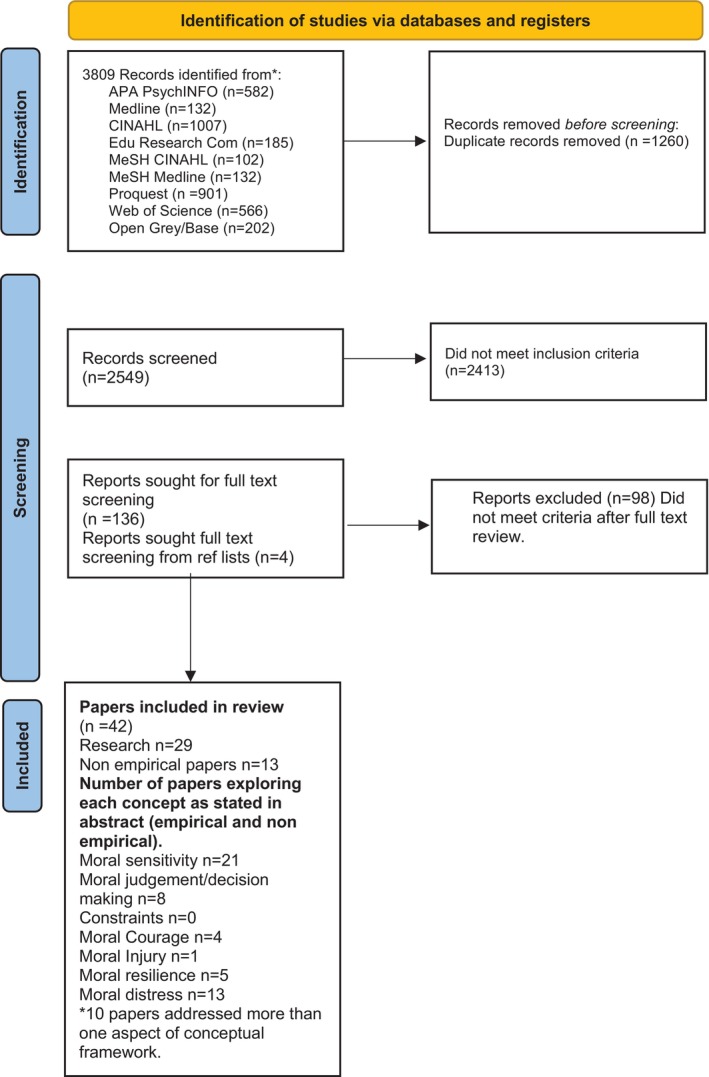
PRISMA 2020 flow diagram for scoping review.

The database and supplementary searching activities returned 3809 records. From these, 1260 duplicates were identified and removed, leaving 2549 papers which underwent title and abstract screening. At this point, 2413 studies were deemed ineligible, and 136 papers underwent full text review. Of those, 42 papers were included in the review; 29 of these were primary research studies and 13 were non‐empirical papers (see Figure 1, PRISMA 2020 flow diagram ScR).

### 3.2 Characteristics of Included Literature

Publishing dates ranged from 2009 (Garity 2009) to 2024 (Ford et al. 2024; Forte et al. 2024; Mattsson 2024; Parker and Welch 2024; Qu et al. 2024; Ziyai et al. 2024). The papers included 4653 student nurses, 30 nurse educators, and 14 health care educators. Moral distress is an international issue, and geographically, papers came from many countries, namely Australia (*n* = 1) (Guzys 2021), Canada (*n* = 3) (Ford et al. 2024; Harvey et al. 2021; Sedgwick et al. 2019), China (*n* = 1) (Qu et al. 2024), Iran (*n* = 9) (Azarkish et al. 2023; Ekramifar et al. 2018; Ghoozlu et al. 2023; Jasemi et al. 2022; Khatiban et al. 2019; Maddineshat et al. 2018; Torabizadeh et al. 2016; Yuksel Kacan 2022; Zia et al. 2023), South Korea (*n* = 4) (Kim and Park 2019; Park 2011; Park et al. 2012; Yeom et al. 2017), Sweden (*n* = 1) (Mattsson 2024), Switzerland (*n* = 1) (Monteverde 2016), Taiwan (*n* = 1) (Lee and Huang 2017), Turkey (*n* = 6) (Baykara et al. 2015; Ertugrul et al. 2022; Kucukkelepce et al. 2020; Nesime and Belgin 2022; Uncu and Gunes 2021; Ziyai et al. 2024), and the United States (*n* = 15) (Ciesielski 2022; Forte et al. 2024; Garity 2009; Howarth 2022; Johansen et al. 2023; Jones‐Schenk and Trepanier 2021; Morrill and Westrick 2022; Parker and Welch 2024; Parsh 2021; Robichaux et al. 2022; Rushton et al. 2017; Townsend and Cummings 2020; Wawersik et al. 2023; Wros et al. 2021; Yoes 2012).

### 3.3 Identified Concepts

In addition to the concepts searched (i.e., moral distress, moral sensitivity, poor patient care, internal constraints, external constraints, moral courage), the review found nurse educators also advocated teaching students about moral decision making, moral injury, and moral resilience in mitigating moral distress. Ten papers stated in the abstract that they were discussing multiple concepts; these included moral courage and moral distress (Ciesielski 2022), moral resilience and moral distress (Forte et al. 2024), moral sensitivity and moral judgement, reasoning, or decision making (Kim and Park 2019; Kucukkelepce et al. 2020; Morrill and Westrick 2022; Park 2011; Park et al. 2012; Ziyai et al. 2024). Two papers discussed moral resilience and moral distress (Rushton et al. 2017; Wros et al. 2021).

The most commonly featured concept that emerged following shortlisting against eligibility criteria was moral sensitivity (*n* = 21; 50%), of which 20 were research papers (Azarkish et al. 2023; Baykara et al. 2015; Ertugrul et al. 2022; Ekramifar et al. 2018; Ghoozlu et al. 2023; Jasemi et al. 2022; Kim and Park 2019; Kucukkelepce et al. 2020; Lee and Huang 2017; Maddineshat et al. 2018; Nesime and Belgin 2022; Park 2011; Park et al. 2012; Qu et al. 2024; Sedgwick et al. 2019; Uncu and Gunes 2021; Yeom et al. 2017; Yuksel Kacan 2022; Zia et al. 2023; Ziyai et al. 2024) and one non‐empirical paper (Harvey et al. 2021). Moral distress featured in 13 (30.9%) papers, of which three were research studies (Ciesielski 2022; Howarth 2022; Forte et al. 2024), and 10 non‐empirical papers (Garity 2009; Guzys 2021; Harvey et al. 2021; Jones‐Schenk and Trepanier 2021; Parker and Welch 2024; Parsh 2021; Robichaux et al. 2022; Rushton et al. 2017; Wros et al. 2021; Yoes 2012). Eight (19.1%) papers emerged featuring the concept moral judgement and decision‐making interventions, of which seven were research papers (Khatiban et al. 2019; Kim and Park 2019; Kucukkelepce et al. 2020; Park 2011; Park et al. 2012; Torabizadeh et al. 2016; Ziyai et al. 2024), and one non‐empirical paper (Morrill and Westrick 2022).

**FIGURE 2:**
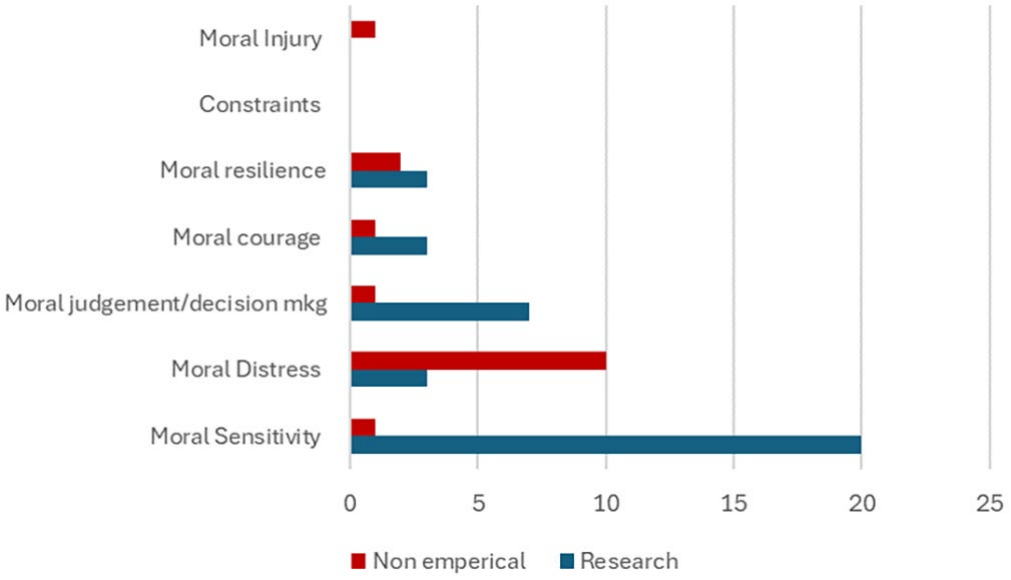
Number of research and non‐empirical papers per concept.

Educating students about moral resilience to mitigate moral distress featured in five (11.9%) papers, of which three were research papers (Forte et al. 2024; Monteverde 2016; Townsend and Cummings 2020), and two were non‐empirical papers (Rushton et al. 2017; Wros et al. 2021). Moral courage featured in four (9.5%) papers, of which three were research papers (Ciesielski 2022; Ford et al. 2024; Wawersik et al. 2023) and one non‐empirical paper (Mattsson 2024). Moral injury featured in one non‐empirical paper (2.3%) exploring this topic (Johansen et al. 2023). Constraints did not feature as the focus of any papers. However, one research paper exploring moral sensitivity (Kim and Park 2019), two research papers exploring moral resilience (Townsend and Cummings 2020; Monteverde 2016), and one non‐empirical paper exploring moral courage, distress, and resilience (Wros et al. 2021) covered constraint content (see Figure 2).

### 3.4 Study Designs

Out of 29 primary research studies, moral sensitivity featured in the highest number of studies (*n* = 20; 68.9%) and adopted a quantitative design in 17 papers. A quasi‐experimental design was seen most frequently and accounted for 10 studies (Azarkish et al. 2023; Ekramifar et al. 2018; Jasemi et al. 2022; Kim and Park 2019; Kucukkelepce et al. 2020; Lee and Huang 2017; Maddineshat et al. 2018; Qu et al. 2024; Yeom et al. 2017; Yuksel Kacan 2022). A randomised controlled trial (RCT) design was utilised in four studies (Baykara et al. 2015; Ertugrul et al. 2022; Nesime and Belgin 2022; Zia et al. 2023), whilst a cross‐sectional design was used in two papers (Park 2011; Park et al. 2012). A descriptive case–control study design was used in a study by Uncu and Gunes (2021). Mixed methods designs were employed in two papers, but their designs were different. Sedgwick et al. (2019) used a convergent parallel design, and Ziyai et al. (2024) used a nested mixed design. One study adopted a qualitative descriptive study design (Ghoozlu et al. 2023).

The other nine studies (31.0%) focused their research instead on moral courage (Ciesielski 2022; Ford et al. 2024; Wawersik et al. 2023), moral judgement and decision making (Khatiban et al. 2019; Torabizadeh et al. 2016), moral resilience (Forte et al. 2024; Monteverde 2016; Townsend and Cummings 2020), and moral distress (Howarth 2022). Research designs included quasi‐experimental designs (Khatiban et al. 2019; Torabizadeh et al. 2016), cross‐sectional survey designs (Ford et al. 2024), and a quantitative descriptive study (Monteverde 2016). There was one mixed methods study using an exploratory design (Forte et al. 2024). Qualitative designs featured three descriptive studies (Ciesielski 2022; Howarth 2022; Wawersik et al. 2023), and one Grounded Theory design (Townsend and Cummings 2020).

### 3.5 Overview of Content, and TALA Over All Seven Concepts Influencing Moral Distress

**FIGURE 3:**
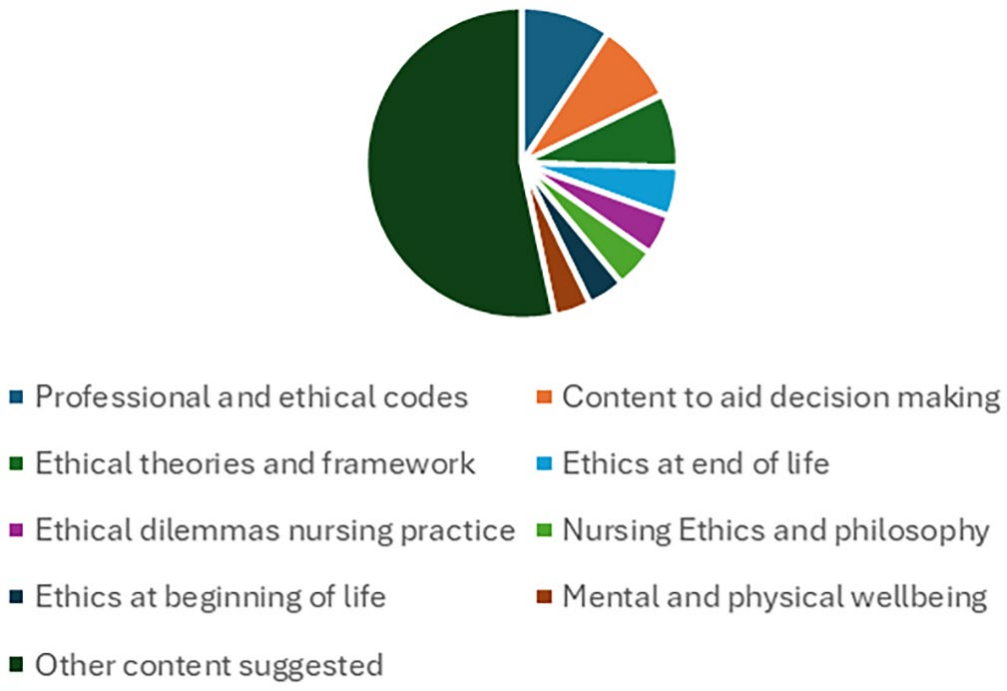
Most frequent overall content subjects.

The review mapped 236 educational content propositions to 70 subject codes (see Table 3 for basic codes and Data S3). The most frequently suggested topics related to the concepts were professional and ethical codes (*n* = 22), content to aid ethical decision making (*n* = 20), ethical theories, frameworks, and principles (*n* = 18), ethics at end of life (*n* = 12), ethical dilemmas for nursing practice (*n* = 10), nursing ethics or philosophy (*n* = 10), ethics at beginning of life (*n* = 9), and physical and mental health and wellbeing (*n* = 9) (see Figure 3).

**FIGURE 4:**
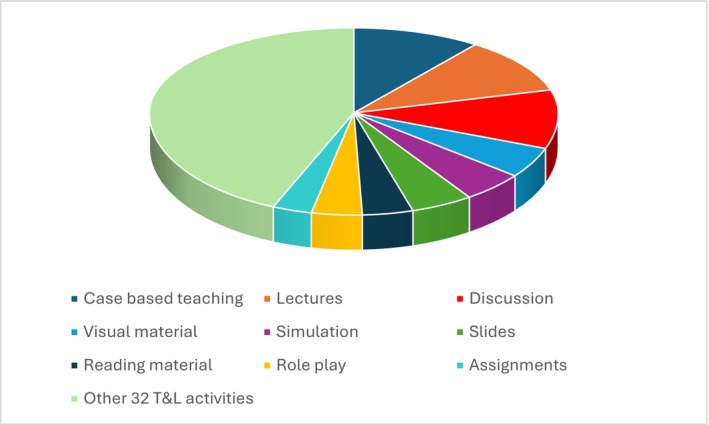
Most frequent overall teaching and learning activity types.

In terms of TALA, 217 propositions were mapped to 41 TALA codes. The most frequently suggested TALA that appeared were the use of case based teaching and learning (i.e., case study, vignettes), (*n* = 25), followed by lectures (*n* = 22), discussion (*n* = 20), reflection (*n* = 11), simulation (*n* = 11), visual material (i.e., video/movie) (*n* = 11), the use of slides to teach content (*n* = 10), role play (*n* = 9), debriefing (*n* = 8), reading material (*n* = 8), and role modelling (*n* = 3) (see Figure 4).

### 3.6 Overview of Content and TALA Per Concept

#### 3.6.1 Moral Sensitivity Content and TALA

Moral sensitivity was the most discussed construct, with 50% of publications exploring the topic; 92 topics were advocated and mapped to 35 subject codes. Professional and ethical code content was referred to most frequently, and in (*n*= 10) (Azarkish et al. 2023; Baykara et al. 2015; Ertugrul et al. 2022; Jasemi et al. 2022; Kim and Park 2019; Maddineshat et al. 2018; Morrill and Westrick 2022; Park 2011; Yeom et al. 2017; Zia et al. 2023); however, the focus of the papers was to evaluate the method of teaching rather than the content provided. Content to aid ethical decision making featured in eight papers (Ertugrul et al. 2022; Kim and Park 2019; Lee and Huang 2017; Maddineshat et al. 2018; Park 2011; Yeom et al. 2017; Zia et al. 2023; Ziyai et al. 2024). However, the approaches to doing this when stated were varied, with some advocating Decision‐Making Guidelines for Licensed Practical Nurses in Canada (Maddineshat et al. 2018) and others citing the Four Topic Approach (Lee and Huang 2017). Ethical frameworks and theories were reported in seven papers (Baykara et al. 2015; Ghoozlu et al. 2023; Kim and Park 2019; Kucukkelepce et al. 2020; Park 2011; Uncu and Gunes 2021; Yeom et al. 2017), but when reported, their philosophical origins varied and emerged from Aristotle, Socrates, Utilitarianism, Duty‐Based, Rights‐Based, and Virtue‐Based ethics (Kim and Park 2019; Park 2011; Yeom et al. 2017), whilst ethical principles were referred to in two papers (Kim and Park 2019; Kucukkelepce et al. 2020).

Nursing philosophy or ethics ranked next with seven papers referring to this subject (Ertugrul et al. 2022; Jasemi et al. 2022; Kucukkelepce et al. 2020; Lee and Huang 2017; Maddineshat et al. 2018; Park 2011; Yeom et al. 2017). Papers referred to content to aid ethical dilemmas in nursing practice (Baykara et al. 2015; Ghoozlu et al. 2023; Park 2011; Qu et al. 2024; Sedgwick et al. 2019; Yeom et al. 2017). Content included the implications of escalation of care to improve patient outcomes (Qu et al. 2024), the impact of poor preoperative (Qu et al. 2024) and post procedure checks (Ghoozlu et al. 2023), truth telling and confidentiality; content was also provided (Qu et al. 2024), as was patient advocacy (Qu et al. 2024) and the student's role in effective cardiopulmonary resuscitation and resuscitation calls (Ghoozlu et al. 2023). Whilst bioethics content was reported in the text once (Maddineshat et al. 2018), typical bioethics content was seen to feature more implicitly when teaching ethics and law, at the beginning of life, where nursing students considered the newborn's right to life (Kucukkelepce et al. 2020), human cloning (Kim and Park 2019), the ethics of abortion (Yeom et al. 2017), discussing euthanasia vs. non‐euthanasia (Yeom et al. 2017), and the ethics of organ donation and transplantation (Kim and Park 2019; Yeom et al. 2017).

Whilst the review found 102 TALA were cited for teaching moral sensitivity, these were mapped to 30 coded activities. The use of case teaching featured most frequently and in 14 papers (Baykara et al. 2015; Ertugrul et al. 2022; Ghoozlu et al. 2023; Kucukkelepce et al. 2020; Lee and Huang 2017; Maddineshat et al. 2018; Morrill and Westrick 2022; Nesime and Belgin 2022; Park 2011; Qu et al. 2024; Uncu and Gunes 2021; Yeom et al. 2017; Yuksel Kacan 2022; Ziyai et al. 2024), but varied in the content within the case, with some exploring ethical violations (Baykara et al. 2015) and others advocacy (Nesime and Belgin 2022). Some cases were conducted in ethical laboratories (Ertugrul et al. 2022), and others in classrooms (Kucukkelepce et al. 2020). The next most frequent TALA occurred via lectures in 11 papers (Azarkish et al. 2023; Ekramifar et al. 2018; Jasemi et al. 2022; Kim and Park 2019; Kucukkelepce et al. 2020; Maddineshat et al. 2018; Morrill and Westrick 2022; Park 2011; Park et al. 2012; Qu et al. 2024; Yeom et al. 2017). However, some lectures were recorded (Azarkish et al. 2023), while some were taught in person (Jasemi et al. 2022). More interactive TALA followed with student discussions in nine papers (Ertugrul et al. 2022; Ekramifar et al. 2018; Lee and Huang 2017; Morrill and Westrick 2022; Park 2011; Park et al. 2012; Uncu and Gunes 2021; Yeom et al. 2017; Yuksel Kacan 2022), and visual material advocated in eight papers (Azarkish et al. 2023; Ertugrul et al. 2022; Ghoozlu et al. 2023; Nesime and Belgin 2022; Qu et al. 2024; Yeom et al. 2017; Yuksel Kacan 2022; Ziyai et al. 2024). Whilst lectures feature in 11 papers, slides, often used to accompany lectures, were reported only in seven papers (Azarkish et al. 2023; Ekramifar et al. 2018; Maddineshat et al. 2018; Morrill and Westrick 2022; Nesime and Belgin 2022; Qu et al. 2024; Zia et al. 2023).

Other TALA that featured less frequently but exclusively relates to moral sensitivity, incorporated the use of mobile phone technology (Azarkish et al. 2023; Maddineshat et al. 2018), was described as project work (Ertugrul et al. 2022; Lee and Huang 2017), and incorporated interactive strategies such as action planning (Nesime and Belgin 2022), concept maps (Azarkish et al. 2023), field visits (Maddineshat et al. 2018), and the use of games to teach ethics (Maddineshat et al. 2018).

#### 3.6.2 Moral Distress Content, and TALA

30.9% of all the literature identified explored interventions to mitigate moral distress. The publications identified 55 topics coded to 32 subject codes. Self‐care and wellbeing strategies featured most frequently and in six papers exploring moral distress (Harvey et al. 2021; Jones‐Schenk and Trepanier 2021; Parker and Welch 2024; Parsh 2021; Wros et al. 2021; Yoes 2012). This topic was ranked highest in this concept than any other. Wellbeing interventions ranged from a wellbeing phone call checking in with students (Jones‐Schenk and Trepanier 2021), giving advice on maintaining adequate hydration and nutrition (Yoes 2012), and mindfulness‐based stress reduction (Parsh 2021; Wros et al. 2021). Code Lavender, a crisis intervention tool, is also proposed and enables words of support, lavender essential oils, and spiritual care if appropriate (Parsh 2021). University professional counselling services for students have also been highlighted in a paper by Parker and Welch (2024) recognising that faculty can facilitate this referral for students.

Reference to professional and ethical codes was highly prevalent and reported in five papers (Garity 2009; Guzys 2021; Parker and Welch 2024; Robichaux et al. 2022; Wros et al. 2021). Content to aid ethical decision making also featured often (Ciesielski 2022; Garity 2009; Harvey et al. 2021; Robichaux et al. 2022). Whilst some did not specify a particular decision‐making model (Harvey et al. 2021), others advocated a range of models, including Bandman and Bandman (1990), Burkhardt and Nathaniel (2008), and Crisham (1992) (Garity 2009).

Nurse educators supported the use of decision‐making content to aid decision‐making skills (Ciesielski 2022; Garity 2009; Harvey et al. 2021; Robichaux et al. 2022), and presented dilemmas for nursing practice (Ciesielski 2022). Ethical theories and frameworks were also advocated, and ranged from ethical principles (Guzys 2021) to Bioethics (Yeom et al. 2017), whilst others included a wider range incorporating Aristotle, Socrates, Altruism, Duty‐Based, Rights‐Based, Virtue‐Based, and Utilitarianism (Garity 2009).

Two frameworks were reported to support student nurses experiencing moral distress. The American Association of Critical Care Nurses (AACN) “4 A's” (i.e., Ask, Affirm, Assess, and Act) Model to Rise Above Moral Distress was referred to in three papers (Parker and Welch 2024; Parsh 2021; Yoes 2012) and received the highest count of content in this concept, despite this being developed for Critical Care Nurses. The second framework was the RAISE programme presented by Parker and Welch (2024), which aids student nurses in recognising and action planning in how to deal with moral distress. Nurse educators also recognised the need for advocacy skills (Guzys 2021; Robichaux et al. 2022; Wros et al. 2021) and conflict resolution content (Robichaux et al. 2022; Yoes 2012).

Despite a wealth of content suggested by nurse educators to mitigate moral distress, two studies cite nurse educators who believed moral distress was not covered in the formal curriculum on which they taught (Ciesielski 2022; Howarth 2022). Whilst nurse educators supported students with moral distress via the unwritten hidden curriculum, they felt they did not adequately prepare students for moral distress and made recommendations to improve moral distress through formal education and adequate clinical placements for students (Ciesielski 2022; Howarth 2022).

There were 36 TALA proposed by nurse educators, mapped to 20 different coded activities. In contrast to moral sensitivity and the overall picture of TALA, nurse educators most frequently advocated debriefing (Harvey et al. 2021; Parsh 2021; Robichaux et al. 2022; Wros et al. 2021), discussions (Ciesielski 2022; Howarth 2022; Jones‐Schenk and Trepanier 2021; Robichaux et al. 2022), and reflection (Ciesielski 2022; Parker and Welch 2024; Robichaux et al. 2022; Wros et al. 2021). Debriefing and reflection featured higher in moral distress than any other concept, and when facilitating reflection, attention was given to the learning environment, emphasising the importance of safe spaces (Wros et al. 2021). Safe spaces enabled regular and intentional outlets for students to reflect on their morally challenging experiences, normalise their feelings, find support for “doing the right thing”, and consider ways to manage moral distress (Wros et al. 2021).

Whilst role modelling was evident across six concepts, it received the highest count in addressing moral distress and emerged in three papers (Parker and Welch 2024; Robichaux et al. 2022; Wros et al. 2021). Distinctive TALA were also advocated in this concept. The proposal of journal clubs (Garity 2009) and ethics consultations (Parker and Welch 2024) was only advocated in moral distress mapped papers.

#### 3.6.3 Moral Judgement and Decision‐Making Content, and TALA

Interventions addressing moral judgement and decision making to mitigate moral distress were identified in 19.1% of the literature included in the review. We found 29 suggestions of content from nurse educators mapped to 16 coded subjects. Like that of moral sensitivity, and the overall picture of educational content, professional and ethical codes featured most often within this concept (Khatiban et al. 2019; Kim and Park 2019; Morrill and Westrick 2022; Park 2011), and appeared alongside content to aid ethical decision making (Khatiban et al. 2019; Kim and Park 2019; Park 2011; Ziyai et al. 2024). However, decision making content was varied, with some not stating a particular model being used (Khatiban et al. 2019), whereas others used the Value‐Be‐Do decision making model (Kim and Park 2019). Ethical theories, frameworks, and principles were featured next most frequently (Kim and Park 2019; Kucukkelepce et al. 2020; Park 2011; Torabizadeh et al. 2016).

Interesting use of educational theory was used to structure content in this concept. In aiding moral development, Morrill and Westrick (2022) used Rest and Narvaez (1994) Theory of Moral Development as a framework for structuring curriculum content to aid ethical decision making but used affective domain TALA (Anderson and Krathwohl 2001) as opposed to cognitive domain (Bloom and Krathwohl 1956) activities.

There were 31 TALA mapped to 16 activity codes. Lecture‐based activities featured highest in teaching students about this concept (Khatiban et al. 2019; Kim and Park 2019; Kucukkelepce et al. 2020; Morrill and Westrick 2022; Park 2011; Park et al. 2012; Torabizadeh et al. 2016). Some used lectures to address cognitive domain objectives, followed by affective domain objective activities (Morrill and Westrick 2022), and as a starting point for discussion in subsequent sessions (Torabizadeh et al. 2016). Effective teaching strategies were cited as including case teaching, discussion, and reflection combined (Morrill and Westrick 2022). Case teaching (Kucukkelepce et al. 2020; Morrill and Westrick 2022; Park 2011; Ziyai et al. 2024) featured within the context of problem‐solving jigsaw learning (Ziyai et al. 2024), and as part of activities designed to achieve affective domain learning objectives (Morrill and Westrick 2022; Park 2011; Park et al. 2012). Whilst problem‐based learning activities were accounted for in only two papers (Khatiban et al. 2019; Park 2011), these instances accounted for a third of all uses of problem‐based learning in the review.

#### 3.6.4 Moral Resilience Content, and TALA

Interventions addressing moral resilience to mitigate moral distress were mentioned in 11.9% of included literature and featured 34 recommendations of content mapped to 26 subject codes. In contrast to any other concept, the subject of ethical theories, frameworks, or principles received the highest ranked content in enabling learning of moral resilience, which was not seen in any other concept (Forte et al. 2024; Monteverde 2016; Rushton et al. 2017). Whilst a symposium of 45 stakeholders has given recommendations that prelicensure curricula include basic ethics content to foster moral resilience (Rushton et al. 2017), ethics content appears to be varied when teaching moral resilience to pre‐licence student nurses. Content advocated in the literature has considered the four ethical principles (Forte et al. 2024), maternal drug abuse (Townsend and Cummings 2020), comfort measures in a patient at end of life with AIDS (Townsend and Cummings 2020), organ donation and transplantation (Wros et al. 2021), and bioethical principles (Wros et al. 2021). In distinguishing between morally wrong and morally complex situations, Monteverde (2016) used vignettes influenced by students' narratives of morally stressful situations. These included the force feeding of an elderly person due to understaffing, and involuntary restraint and medication of a psychotic patient (Monteverde 2016).

Content distinctive to moral resilience was suggested to include compassion fatigue (Townsend and Cummings 2020), incivility (Townsend and Cummings 2020), sensory awareness skills (Forte et al. 2024), and the use of naming, framing, and claiming frameworks (Forte et al. 2024), enabling individuals to name the ethical issue, frame the perspectives of all stakeholders, and claim their voice by addressing the issue (Forte et al. 2024).

In total, 21 TALA related to moral resilience were mapped to 17 coded activities. While didactic (Forte et al. 2024) and lecture‐based (Monteverde 2016; Townsend and Cummings 2020) teaching strategies to foster moral resilience were used often, interactive methods such as case teaching (Monteverde 2016; Wros et al. 2021) was advocated but varied in their content. Debriefing (Sedgwick et al. 2019; Wros et al. 2021) and simulation were also noted to be proposed as frequently (Forte et al. 2024; Townsend and Cummings 2020).

#### 3.6.5 Moral Courage Content, and TALA

Of all included papers, 9.5% were mapped to interventions cultivating moral courage. This yielded 20 suggestions of content mapped to 18 content codes. The range of topics were quite broad and lower in counts per subject; however, unique content was proposed in fostering moral courage. Implicit bias and conscientious objection (Ford et al. 2024), defined by the International Council of Nurses (2021) as the refusal to participate in required action, or seeking exemption from participation in classes of interventions (e.g., abortion, gender reassignment surgery, organ transplantation) that threaten a person's sense of moral integrity, were viewed as a priority topic in nursing ethics education (Ford et al. 2024). Others emphasized basic nursing skills knowledge which educated students to address poor nursing care they witness in practice settings (Mattsson 2024). The importance of ethics teaching in the gerontological populations is advocated on the basis that the aging population is most engaged with nursing (Mattsson 2024). Team working was proposed using team Strategies and Tools to Enhance Performance and Patient Safety (TeamSTEPPS) (Wawersik et al. 2023).

There were 17 teaching and learning proposals mapped to 12 coded activities. Reflection (Ciesielski 2022; Ford et al. 2024; Mattsson 2024; Wawersik et al. 2023) and discussion (Ciesielski 2022; Wawersik et al. 2023; Mattsson 2024) received the highest count of educational strategies, but one paper mentioned that this discussion often took place in the hidden curriculum (Ciesielski 2022), whereas other proposals included peer‐to‐peer discussion (Wawersik et al. 2023) and online discussions (Mattsson 2024). Although receiving a lower count, salient activities emerged when educators considered the learning environment. Nurse educators enabled moral spaces, which allowed students to articulate concerns and feel supported when moral courage was required, a strategy to cultivate courage (Ford et al. 2024). Brave spaces, in contrast to moral spaces, allowed the student to lean into discomfort and place emphasis on genuine peer learning, which was deemed to be a more fitting approach to discussing challenging topics in nursing ethics courses (Ford et al. 2024).

The importance and impact of faculty role modelling was also highlighted. Faculty offered opportunities to influence the learning environment when fostering moral courage, by role modelling and acknowledging they too have made mistakes, which shows vulnerability and humanises the instructor (Wawersik et al. 2023).

#### 3.6.6 Moral Injury Content and TALA

Whilst moral injury received attention from one paper (Johansen et al. 2023), remarkable content was proposed that did not feature in any other concept. Content relating to natural disasters (e.g., pandemics) and post‐traumatic stress disorder (PTSD) was advocated by Johansen et al. (2023).

Johansen et al. (2023) propose at least 10 different coded TALA (i.e., case studies, debriefing, lecturing, simulation, and role modelling). An innovative approach unique to moral injury, however, was not just teaching self‐care but the recommendation of the self‐care assignment detailing how the student would perform with self‐care (Johansen et al. 2023).

#### 3.6.7 Constraints

There were no papers where the title or abstract identified that the focus of the paper was to investigate internal or external constraints. Some content on constraints, however, did feature in the literature, but this occurred in papers where the focus was to explore other concepts, i.e: moral sensitivity (Kim and Park 2019), moral resilience (Monteverde 2016; Wros et al. 2021), and moral distress (Wros et al. 2021).

External constraints as described by Jameton (1993) were portrayed by Monteverde (2016) who used vignettes to illustrate a patient being force fed due to a lack of staffing. Wros et al. (2021) also portray external constraints when discussing the case of a refugee being recommended a lung transplant by a pulmonologist, but being declined this due to her poor English, lack of stable income, and inadequate support network.

Topics covering internally constrained distress, characterised by self‐doubt, lack of assertiveness, or lack of understanding (Epstein and Hamric 2009), were covered by nurse educators when covering topics of incivility and workplace bullying (Townsend and Cummings 2020). Psychological safety was also identified (Wawersik et al. 2023) and was taught to encourage the reporting of errors in clinical practice, often shaped by fear and perceptions of disempowerment in students (Timmins 2024), which influences whether these errors are reported or not.

## 4 Discussion

In performing this review, the authors sought to use the findings to inform curriculum development and develop a research agenda. Nurse educators have made significant strides in developing educational interventions addressing moral distress directly, and through concepts indirectly. The review found that educational content and TALA across the influencing concepts, and within each concept was heterogenous in nature, often taking place in different contexts, alongside different interventions with different sub content.

Overall educational content on professional and ethical codes featured most frequently (9.3%) and featured in five out of the seven concepts. Such codes, as seen in the International Council of Nurses (2021) Code of Ethics, are a useful tool to set expectations of professional practice. Whilst no study has explored the impact of teaching nursing students on professional ethical codes and the impact they may have on moral distress, Jasemi et al. (2022) found teaching students Codes of Ethics demonstrated a statistically beneficial difference in the mean score of moral sensitivity immediately after, and 2 months after the interventions of lecture and role play. Azarkish et al. (2023) also highlighted teaching ethical codes to nursing students via flipped classroom methods and Short Message Service (SMS) had a statistically favourable effect upon moral sensitivity following the intervention. Chen et al. (2021) also found when using the Nurses Professional Values Scale—Revised (Gong et al. 2011), derived from the American Nurses Association Code of Ethics (ANA 2001), that demonstrating professional values in line with the ANA Code had a mediating effect on moral sensitivity, also positively correlating with ethical decision‐making.

Ethical decision‐making content (8.5%) featured next most frequently and was proposed by nurse educators across six of the seven concepts. However, educational ethical decision‐making models (EDM), when stated, varied. Georgieva et al. (2024) found 24 EDM were being used in healthcare. Johnson et al. (2021) identified 38 unique EDM used in mental health. To our knowledge, no scoping review or systematic review has been conducted identifying the types and effectiveness of EDM in the student nurse population. Our review found that Jonsen and Winslade (2010) Four‐Topic approach was used to guide nursing students' decision making (Lee and Huang 2017). However, the Four Topic approach is a clinical ethics case analysis approach representing a set of questions considering medical prognosis and quality of life, requiring medical judgment (Schumann and Alfrande 2008) and does not fall within the remit of student nurses' decision making. Further research is needed in this area to investigate appropriate EDM for use in the student nurse population to aid in mitigating moral distress.

Despite ethical theories and principles being referred to on 18 occasions (7.6%), only six studies reported details of the ethics content that was advocated. Ethical theories and principles varied and ranged from Utilitarianism, Deontology, Virtue Ethics, and Principlism Ethics. There are, however, critics of using normative ethical theories for clinical nursing practice (Beauchamp and Childress 2019). Some suggest normative ethics are instructive and instead contribute to our thinking about moral life and not nursing practice (Beauchamp and Childress 2019). Whilst ethical rules and principles guide ethical conduct, Noddings (1984) highlights how they frequently function in a manner destructive of the very relationships that an ethics of care, very relevant in nursing, seeks to preserve.

The value foundations of nursing ethics are derived from the nature of the nurse–patient relationship instead of from models of patient good, rights‐based notions of autonomy, or the social contract of professional practice as articulated in prominent theories of medical ethics (Fry 1989). Engaging student nurses in medical‐oriented ethics requires acknowledgement of the nurse's moral agency, which is their notion of right and wrong and is held accountable for their actions (Traudt et al. 2016), and is enabled by situational, contextual, and structural features of the moral terrain (McCarthy and Gastmans 2015). Failing to recognize the student nurse's moral agency may render student nurses perceiving they are, mistakenly, decision makers in situations when they are taught about medical ethical issues; that is, choices relating to the newborn's right to life, and could instead generate moral distress, due to being constrained to act upon their judgment of right or wrong in this instance.

The overall most frequently advocated TALA involved the use of case‐based learning (11.5%) and was commonly referred to when exploring moral sensitivity. However, all studies using case‐based learning as an intervention in addressing moral sensitivity did so in conjunction with other bundles of teaching activities to facilitate student learning and varied with how the case was presented. In line with other literature, Yao et al. (2023) found in their systematic review of mixed studies that there is no common format for the case design and case‐based learning implementation process. It may be prudent, however, to incorporate cases that have been found challenging to student nurses and which generate moral distress, as found in a concept analysis of moral distress in students by Timmins (2024). Case‐based learning is reported to be advantageous as an active learning approach, encouraging engagement with the case by the learner. Taylor et al. (2023), in a cross‐sectional study of 717 nursing students, identified high engagement with online case studies and reported factors that contributed to this was preparation for their clinical placement; and a higher likelihood of high self‐efficacy.

Lectures were the second most frequent TALA (10.1%) overall addressing the concept of moral distress but featured more prominently when exploring moral sensitivity as part of combined interventions. Whilst lectures are considered a didactic and less interactive method of teaching, they continue to have a role in Higher Education. When used, lectures were effective in significantly increasing students' moral sensitivity (Jasemi et al. 2022). However, when evaluated for correct ethical decision‐making in nursing students, and in comparison to problem‐based learning, neither of the methods significantly increased the rate of correct decision‐making (Khatiban et al. 2019).

## 5 Strengths/Limitations

Strengths are the review was conducted in line with JBI guidelines for scoping reviews (Aromataris et al. 2024; Peters et al. 2020). A comprehensive search of published literature and grey literature was conducted to address the research questions. The first author has subject expertise in moral distress, is an educator teaching pre‐registration student nurses, and has curriculum development experience. The second author is an experienced researcher proficient in conducting scoping reviews.

Potential limitations to the review are that it excluded papers not written in the English language. In addition, the protocol was also not shared beyond the supervisory team. Whilst scoping reviews may be considered a precursor to a systematic review, critical appraisal of the included studies was not conducted and is not required in scoping reviews. Bias was managed by using a basic coding scheme and strategies such as member checking, reflexivity, and peer debriefing (Lincoln and Guba 1985).

Limitations to the evidence in the review were noted to include content, and TALA were not always stated in detail. Some studies omitted reporting important content and did not use reporting criteria for their studies, influencing the quality of scholarship. Ten papers also addressed multiple concepts (i.e., moral sensitivity, courage, judgement, moral distress, and resilience) and were unclear which content or TALA were related to which concept and are therefore mapped to both, which could influence the results in these concepts.

## 6 Conclusion

A research agenda addressing educational research to improve ethical nursing practice and including moral distress and pre‐licence student nurse curriculum (Koirala et al. 2022; Rushton et al. 2017) is noted. In addition to the agenda, this review adds several recommendations for investigating how nurse education can equip pre‐registration student nurses with evidence‐based interventions to mitigate moral distress.

Firstly, in developing educational research in this area, reporting guidelines for educational research studies should be developed. The review found four RCTs included in this review; however, only one study (Nesime and Belgin 2022) reported the use of any type of reporting guidelines (e.g., CONSORT) and pre‐registered their review protocol. During the review, it was also noted that important content was missing in RCTs; for example, comparators or interventions were not fully disclosed, rendering it difficult to fully interpret the findings or have full confidence in the findings. Developing reporting guidelines would aid evidence‐based investigation of moral distress educational research to remedy the omission of key information, enabling transparency and improving the quality of scholarship in this area (O Brien et al. 2020). In doing this, it aids the development of evidence‐based research essential to facilitate the trajectory of evidence toward improving future related activity (Aromataris et al. 2024).

Secondly, the review acknowledges that educational research across the concepts derives largely from the concept of moral sensitivity. Recommendations are made to develop educational research across all concepts influencing moral distress. Multicomponent educational interventions exist across all influencing concepts; however, their direct impact on moral distress is unknown. Recommendations are made to explore how educational interventions addressing one concept influence moral distress. There was overlap in educational content and TALA across all the concepts. Recommendations are made, therefore, to identify and map which content is best suited to address which concept and what TALA are best suited to facilitate that content.

It is acknowledged that educators often implement multiple methods of teaching and learning activities to address different learning styles and objectives, however, this challenges development of evidenced based educational interventions to address moral distress. It would seem plausible to conduct primary research studies to investigation the impact of single educational interventions across all the concepts claimed to influence moral distress and investigate the impact of this on nursing student's moral distress. Once the effectiveness of these interventions is known, combining bundles of interventions and evaluating the impact could be considered next. A systematic review critically appraising single component educational interventions and a critical appraisal of the evidence on moral distress in student nurses would also be welcomed.

## Conflicts of Interest

The authors declare no conflicts of interest.

## Supporting information


Data S1.

Data S2.

Data S3.

## Data Availability

Yes our data is available from our scoping review. The data that supports the findings of this study are available in the Supporting Information of this article. Any additional data can be requested from the authors.
