# Supplementary material for: Educational Strategies for Managing Moral Distress in Student Nurses: A Scoping Review
Source: J Adv Nurs. 2025 Nov 3;82(6):5701–26. doi: 10.1111/jan.70320 (PMC13176732; doi:10.1111/jan.70320)
Supplement: Supplementary file 1 — Data S1. [file JAN-82-5701-s003.docx]

**Scoping Review Protocol**

**Educational Strategies for Managing Moral Distress in Student Nurses: A Scoping Review.**

**by**

**Rebecca Timmins^[[1]](#footnote-1)^ and Dr Chris Kite^[[2]](#footnote-2)^**

**Abstract:**

**Background:** Student nurses experience moral distress in clinical practice. Nurse educators have a role in preparing student nurses for clinical practice.

**Aim**: To explore what content, teaching and learning activities are advocated by nurse educators to mitigate moral distress and associated concepts in student nurses.

**Design**: This scoping review protocol follows the Joanna Briggs Institute (JBI) methodological guidance for scoping reviews and Preferred Reporting Items for Systematic reviews and Meta-Analyses extension for Scoping Reviews (PRISMA-ScR) Checklist. Eligibility criteria reflected the Population, Concept, Context (PCC) format.

**Methods**: A search strategy is proposed and included in the protocol using both Medical Subject Headings (MeSH) and a researcher designed strategy using PCC format. JBI 3 step search strategy is planned. Database searches will include CINAHL Ultimate, MEDLINE Full Text, APA PsycINFO, Education Research Complete, Web of Science, ProQuest, Base and Open Grey. Search limiters include English language, but no restrictions on publication age, and considers empirical and non-empirical papers. The protocol identifies a comprehensive search strategy, inclusion criteria based upon PCC format, and identifies provisions for data extraction, data charting, synthesis and analysis.

**Impact:** This is an important first step in firstly identifying educational content, and teaching and learning activities aimed at mitigating moral distress, and the concepts influencing moral distress, in order to develop a comprehensive curriculum for student nurses. The scoping review has the potential to direct future educational research on the topic.

**Introduction**

Moral distress was first introduced by Jameton (1984) who following encounters with student nurses identified that moral distress occurs when one knows the right thing to do, but institutional constraints, and/or conflicts with co-workers make it impossible to pursue the right course of action. The negative impact of moral distress in student nurses is evidenced in papers by Bickhoff et al., (2017) and Sasso et al., (2016), who recognise negative physical and psychological problems for the student. Studies also highlight the student’s increased risk of suicide and depression (Paidipati et al., 2023), frustration, and guilt (Escolar Chua et al., 2019 ; Sasso et al., 2016), with some so affected, they wanted to leave the profession (Yilmaz et al., 2022). A negative impact on patient care can also result due to students remaining silent and not acting upon morally reprehensible events they witness in clinical practice (Bickhoff et al., 2017; Yilmaz et al., 2022).

Prior to conducting this review, a thorough review of published and unpublished literature was conducted to identify a scoping review or systematic review examining educational interventions in the student nurse population with the aim of mitigating moral distress. The Cochrane library, Joanna Briggs systematic reviews, PROSPERO, PubMed, Web of Science resources were searched but no scoping review or systematic review were found to exist on the subject. Several other papers, however, identified there had been some development in addressing moral distress through interventions to mitigate moral distress, but not in the student nurse population.

A scoping review by Chen et al., (2024) identified nine papers which described and evaluated the effectiveness of interventions in paediatric Intensive Care Unit (ICU) nurses. A systematic review by Zeydi et al., (2022) identified eight interventions being evaluated in critical care registered nurses. Also, 22 interventions were analysed in the Registered Nurse population (Amos et al., 2022). A systematic review by Morley et al., (2021) explored 16 papers disclosing interventions to mitigate moral distress, however, only one study was discussed in the population of student nurses (Monteverde, 2016). However, interventions that are effective in mitigating moral distress the Registered Nurse population, may be ineffective in the student nurse population due to students experiencing moral distress distinctively (Timmins, 2024).

The systematic review by Morley et al., (2021) noted educational interventions were often multifaceted and took the form of a variety of different bundles of different interventions, due to the diffuse nature of moral distress. However, it was unclear which intervention had what effect on moral distress (Morley et al., 2021). Whilst Meng et al., (2025) conducted a systematic review of interventions to improve the ethical sensitivity of student nurses, Timmins (2024) found conceptually, the attributes of moral distress in student nurses are multifaceted and consist not only of moral sensitivity, but is influenced by other concepts such as the root causes of moral distress, internal constraints, external constraints, and moral courage. Therefore, in developing a comprehensive educational programme to mitigate moral distress in student nurses, it would be prudent to consider how nurse education can also address the interrelated concepts that can influence moral distress occurring to mitigate this pervasive issue. Therefore, for the purposes of the scoping review, when referring to mitigating moral distress, this also included the interrelated concepts influencing this and as seen in the search strategy.

This is an important first step in developing a comprehensive curriculum to mitigate moral distress in student nurses. Nurse educators serve a crucial role in the preparation of nurses and can use teaching strategies to address moral distress during student nurse training (Loyd et al., 2023).

**Keywords:** Education; ethics; intervention; student nurse; moral distress

**Aims**

This scoping review aims to examine the scope, and nature of interventions, advocated by nurse educators to mitigate the negative effects of moral distress in student nurses.

The objectives of the scoping review are to:

1. Identify what content is advocated by nurse educators to mitigate moral distress and interrelated concepts influencing this.
2. Identify what teaching and learning activities are advocated by nurse educators to mitigate moral distress and interrelated concepts influencing this.
3. Examine how advocated content, and teaching and learning activities align with moral distress and interrelated concepts.
4. Identify how advocated educational content aligns with the evidence base highlighting how student nurses experience moral distress.
5. Identify how teaching and learning approaches can potentially align to cognitive, affective and psychomotor domains of learning (Bloom et al., 1956, Anderson et al., 2001).
6. Identify how empirical research is conducted on the topic.
7. Identify gaps in knowledge and the existing literature.

**Methodology**

This protocol was developed in line with Joanna Briggs Institute (JBI) Methodology for Scoping Reviews (Peters et al., 2020) and Preferred Reporting Items for Systematic reviews and Meta-Analyses extension for Scoping Reviews (PRISMA-ScR) Checklist( Tricco et al., 2018).

**Research Questions**:

This scoping review protocol uses the Population, Context, Concept (PCC) principles to address the research and to define eligibility criteria (Peter et al., 2020).

The research questions guiding this scoping review protocol are:

1.What educational content is proposed and/or implemented by nurse educators (context) to mitigate the negative impact of moral distress (concept) in student nurses (population)?

2.What teaching and learning activities are proposed and/or implemented by nurse educators (context) to mitigate the negative impact of moral distress (concept) in student nurses (population)?

3. How is research conducted by nurse educators (context) to mitigate moral distress (concept) in student nurses (population)?

4. How could nurse education develop content, and teaching and learning material (context) to mitigate moral distress (concept) in student nurses (population)?

**Inclusion criteria**

**Table 1 Inclusion/Exclusion criteria for ScR.**

| Domain | Inclusion criteria | Exclusion criteria |
| --- | --- | --- |
| Population – student nurses | Student nurses who are on a course leading to professional registration as a Nurse.  Student nurses of all fields of nursing (e.g Adult, Mental Health, Child, Learning Disability, no field specified).  Student nurses on Undergraduate or master’s programmes leading to registration as a nurse.  All genders, age, ethnicity. | Student nurses who are already registered as a nurse on a professional register.  Students on a course about nursing care but does not lead to professional registration as a nurse. |
| Context – Interventions by Nurse Educators | Any content advocated by nurse educators to mitigate moral distress.  Any teaching and learning activities advocated by nurse educators to mitigate moral distress.  Ethics education advocated in the context of mitigating moral distress.  Nurse educators are professional registered nurses who have a role in nurse education.  Nurse educators of all genders, age, ethnicity and of any field of nursing (e.g Children’s nursing, Mental Health, Learning Disability, no field specified). | Content and teaching and learning activities that do not intend to mitigate moral distress.  Ethics education not discussed in the context of mitigating moral distress.  Professional nurses who do not have a role in nurse education.  Individuals who are not professional nurses. |
| Concept - Moral distress and interrelated concepts. | Moral distress.  Moral sensitivity.  Causes of moral distress (e.g poor patient care).  Internal constraints.  External constraints.  Moral courage.  Consequences of moral distress (i.e physical and emotional symptoms). | Concepts not related to moral distress. |
| Sources of literature | Literature in english language.  No age restriction.  Papers from all geographical areas.  Empirical and non-empirical literature.  Grey literature (i.e Thesis and Dissertations). | Books.  Policy documents.  Clinical guidelines.  Papers not in english language. |

**Search Strategy**

The approach for searching published papers follows the same three step method as in standard JBI systematic reviews and included engagement with key stakeholders (i.e University librarian) (Peters et al., 2020). Step 1. An initial limited search in CINAHL Ultimate and Pub Med on topic of interest. The keywords used in the titles and abstracts of identified articles, and index terms will be identified and used to develop a full search strategy (see Table 2 for search terms, Table 3 search strategy). Step 2. Database-specific searches are then conducted in APA Psych INFO, CINAHL, Education Research Complete, Medline according to search terms and information sources selected and included as per inclusion criteria. Step 3. Scanning the reference lists of all studies selected to identify any additional relevant studies.

Grey literature will be searched via Web of Science, ProQuest, Base and Open Grey as per University of Wolverhampton (2024) Guide to Searching Grey Literature. Due to the simplistic search functions in these databases search terms “Moral Distress” AND “Education” will be used. Due to the iterative nature of the search additional keywords, and sources, and potentially useful search terms may be discovered and incorporated into the search strategy (Peter, Marnie, Tricco et al 2020).

The aim of the review does not seek to identify the effectiveness of the intervention; therefore, considers all sources of literature within the hierarchy of evidence (Aveyard et al, 2020). Researcher developed search terms and MeSH Terms were formulated using PCC format.

**Table 2: Search Terms (Researcher developed and MeSH terms )**

| Population | Context | Concept |
| --- | --- | --- |
| Nurs* Student*  Bachelorette  Undergraduate nurs*  Students, Nursing [MeSH] | Intervention  Education  Address  Alleviate  Reduce  Mitigate  Nurse Educat*  Teach*  Tutor  Education Nursing [MeSH]  Nursing Faculty [MeSH]  Nurses [MeSH] | “Moral Distress”  “Moral Stress”  “Ethics stress”  “Ethical Distress”  “Moral Sensitivity”  “Ethical Sensitivity”  “Moral Courage”  “Poor patient care”  “Harm to patient”  “Unsafe care”  “Internal Constraints”  “External Constraints” |

**Table 3: Search Strategy**

| Search Number | Search Terms |
| --- | --- |
| #1  #2  #3  #4  #5  #6  #7  #8  #9  #10  #11  #12  #13  #14  #15  #16  #17  #18  #19  #20  #21  #22  #23  #24  #25  #26  #27  #28  #29  #30  #31  #32 | Nurs* Student* (Title/Abstract)  Bachelorette (Title/Abstract)  Undergraduate nurs* (Title/Abstract)  Students, Nursing [MeSH]  #1OR #2 OR #3 OR #4  Intervention (Title/Abstract)  Education (Title/Abstract)  Address (Title/Abstract)  Alleviate (Title/Abstract)  Reduce (Title/Abstract)  Mitigate (Title/Abstract)  Nurse Educat* (Title/Abstract)  Teach* (Title/Abstract)  Tutor (Title/Abstract)  Education Nursing [MeSH]  Nursing Faculty [MeSH]  Nurses [MeSH]  #6 OR #7 OR #8 OR #9 OR #10 OR #11 OR #12 OR #13 OR #14 OR #15 OR #16 OR #17  “Moral Distress” (Title/Abstract)  “Moral Stress” (Title/Abstract)  “Ethics stress” (Title/Abstract)  “Ethical Distress” (Title/Abstract)  “Moral Sensitivity” (Title/Abstract)  “Ethical Sensitivity” (Title/Abstract)  “Moral Courage” (Title/Abstract)  “Poor patient care” (Title/Abstract)  “Harm to patient” (Title/Abstract)  “Unsafe care” (Title/Abstract)  “Internal Constraints” (Title/Abstract)  “External Constraints” (Title/Abstract)  #19 OR #20 OR #21 OR # 22 OR #23 OR #24 OR #25 OR #26 OR #27 OR #28 OR #29 OR #30  #5 AND #18 AND #31 |

**Search limiters**

Only papers in English language are included in the search strategy. Language restrictions are permitted if there is reasonable justification, such as feasibility or limitation of resources (Peter et al., 2020). Due to the broad nature of the search and researcher participation being restricted to two people, language restrictions are applied. The search strategy does not include any other restrictions on searching (e.g date, Geography or date).

**Study Selection.**

Search results will be exported into the review manager software Rayyan, where duplicates will be removed. JBI guidelines refer to two or more researchers reviewing selection of papers and arrangements for any disagreements (Peter et al., 2020). Having two or more researchers review papers ensures a fair and impartial selection process, further strengthening the review’s reliability (McCleod, 2024). Title and abstract screening will be completed independently by two researchers, and studies which are potentially eligible will be carried forward to a full-text screening. Any disagreements around eligibility will be resolved by discussion with a third researcher.

The scoping review will be conducted by one doctoral student subject expert researcher, and one researcher experienced in conducting scoping reviews.

**Data extraction tool (Charting the results).**

Data extraction fields should align with the research questions (Peters et al., 2015). Therefore, a standardised data extraction form will be created to enable data collection of authors, date of publication, country of origin, aims/purpose, population, sample size, sampling method, study design, intervention type, concept(s) addressed, educational content, teaching and learning activities, outcomes, key findings, and limitations.

**Data Presentation**

Results should be extracted and charted using Preferred Reporting Items for Systematic Reviews and Meta-Analyses (PRISMA) flow diagram for the scoping review process (Peter et al., 2020). A narrative description of the search decision process accompanied by the flow chart will be presented**.** Where data allows a visual representation of key findings will be provided. Data will be presented in Tables and/or Figures related to the scoping reviews research questions, aims and objectives.

**Data Analysis**

Data in this scoping review will be analysed by addressing each research question with a narrative explanation addressing this. Descriptive data will be provided to address the objectives of the review.

**Changes to Protocol**

Unlike systematic review, due to the iterative nature of a scoping review, some changes to protocol may be necessary (Peters et al., 2015). Any discrepancies will be clearly detailed and justified in the ‘Methods’ section of the scoping review and report, if, and when they occur (Peter, et al., 2020).

**Conclusion**

To the best of our knowledge, this is the first scoping review that explores how nurse educators address moral distress in pre-registration nursing. This is an important first step of development of research and evidence-based interventions to address and alleviate the negative effects of moral distress in this population**.**

**Funding:** This protocol forms part of Rebecca Timmins doctoral work investigating moral distress education as part of their Doctoral studies and is funded by University of Wolverhampton, UK.

**References**

Amos V, Epstein E (2022) Moral Distress Interventions: An integrative literature review, Nursing Ethics, 29 (3) 582-607.

Anderson L, Krathwohl D (2001) A taxonomy for learning, teaching, and assessing : A revision of Bloom’s taxonomy of educational objectives. Longman

Bickhoff L, Sinclair P, Levett-Jones T (2017) Moral courage in undergraduate nursing students: A literature review. Collegian, 24(1), pp71–83

Bloom B, Krathwohl D et al. (1956) Taxonomy of educational objectives: The classification of educational goals, by a committee of college and university examiners. Handbook I: Cognitive domain. NY, NY: Longmans, Green

Chen J, Lin N, Ye X, et al (2024) Coping strategies and interventions to alleviate moral distress among pediatric ICU nurses: A scoping review, Nursing Ethics, May 2024, online and ahead of print. doi: 10.1177/09697330241252875

Escolar Chua R, Magpantay J (2019) Moral distress of undergraduate nursing students in community health nursing. Nursing Ethics, 26(7/8), 2340–2350.

Jameton A (1984) Nursing practice: The ethical issues, Englewood Cliffs, NJ: Prentice Hall

Loyd L, Leesberg Stamler L, Culross B (2023) Early career nurses and moral distress: An integrative review, Nurse Educ Pract Nov:73:103844. doi: 10.1016/j.nepr.2023.103844

Meng J, Xu Y (2025) Effective intervention measures to improve the ethical sensitivity of nurses and nursing students, Nurse Education in Practice, Volume 83, February 2025, 10428

McCleod S (2024) Doing a Scoping Review: A Practical, Step-by-Step Guide, Psychology, Methodology https://www.simplypsychology.org/steps-for-conducting-a-scoping-review.html accessed 1/1/25

Morley G, Field R, Horsburgh C et al (2021) Interventions to mitigate moral distress: A systematic review of the literature, International Journal of Nursing Studies, Vol 121 103984

Monteverde S (2016) Caring for tomorrow’s workforce: Moral resilience and healthcare ethics education, Nursing Ethics 23(1) 104–116.

Paidipati, C, Lozano A, West J et al (2023) Understanding the mediated relationship between moral distress, depression, and suicide risk in undergraduate nursing students. Nursing Outlook, 71(3), 101966.

Peters M, Godfrey C, Khalil H et al (2015) Guidance for conducting systematic scoping reviews, International Journal of Evidence-Based Healthcare 13(3):p 141-146

Peters M, Marney C, Tricco A, et al (2020) Updated methodological guidance for the conduct of scoping reviews, JBI Evid Synth 18 (10): 2119-2126.

Sasso L, Bagnasco A, Bianchi M et al (2016) Moral distress in undergraduate nursing students: A systematic review’, Nursing Ethics, 23(5) pp523-34

Timmins R (2024) Student nurses experiences of moral distress: A concept analysis, Journal of Advanced Nursing, 05 August 2024 https://doi.org/10.1111/jan.16370

Tricco A, Lille E, Zarwin W et al (2018) Preferred Reporting Items for Systematic reviews and Meta-Analyses extension for Scoping Reviews (PRISMA-ScR) Checklist, Annals of Internal Medicine Volume 169, Number 7, https://doi.org/10.7326/M18-0850

Yilmaz S, Kiziltepe, K (2022) Moral distress in nursing undergraduates: A qualitative study. Karya Journal of Health Science, 3(2), 62–68.

Zeydi A, Gazanfari M, Suhoen R et al (2022) Effective interventions for reducing moral distress in critical care nurses, Nursing Ethics, 29(4):1047-1065

1. School of Nursing, Faculty of Education, Health and Wellbeing, University of Wolverhampton, City Campus, Wolverhampton, UK. [↑](#footnote-ref-1)
2. School of Health and Wellbeing, Faculty of Education, Health and Wellbeing, University of Wolverhampton, Wolverhampton WV1 1LY, UK.

   Division of Public Health, Sport and Wellbeing, Faculty of Health, Medicine and Society, University of Chester, Chester CH1 4BJ, UK.

   Warwickshire Institute for the Study of Diabetes, Endocrinology and Metabolism (WISDEM), University Hospitals Coventry and Warwickshire NHS Trust, Coventry CV2 2DX, UK. [↑](#footnote-ref-2)
