# Supplementary material for: Educational Strategies for Managing Moral Distress in Student Nurses: A Scoping Review
Source: J Adv Nurs. 2025 Nov 3;82(6):5701–26. doi: 10.1111/jan.70320 (PMC13176732; doi:10.1111/jan.70320)
Supplement: Supplementary file 3 — Data S3. [file JAN-82-5701-s002.docx]

**Supplementary File 3: Basic Coding table of educational content and Teaching and Learning Activities (TALA) where papers are aligned to codes.**

.

| **Moral sensitivity educational content** | | **Moral sensitivity TALA** | |
| --- | --- | --- | --- |
| **Professional and ethical codes** (n=10) (Azarkish et al.,2023; Baykara et al., 2015; Ertugral et al., 2022; Jasemi et al., 2022; Kim et al., 2019; Maddenshatt et al., 2018; Morrill et al., 2022; Park, 2011; Yeom et al., 2017; Zia et al., 2023).  **Content to aid ethical decision making** (n=8) (Ertugral et al., 2022; Kim et al., 2019; Lee et al., 2017; Maddineshat et al., 2018; Park, 2011; Yeom et al., 2017; Zia et al., 2023; Ziyai et al., 2024).  **Ethical Frameworks, theories, principles** (n=7) (Baykara et al., 2015; Ghoozlu et al., 2023; Kim et al., 2019; Kucukkelepce et al., 2020; Park, 2011, Uncu et al., 2021; Yeom et al., 2017).  **Nursing ethics** (n=7) (Ertugral et al., 2022; Jasemi et al., 2021; Kucukkelepce et al., 2020; Lee et al., 2017; Maddenshatt et al., 2018; Park, 2011; Yeom et al., 2017).  **Ethics for nursing practice** (n=6) (Baykara et al., 2015; Ghoozlu et al., 2023; Park, 2011; Qu et al., 2024; Sedgewick et al., 2019; Yeom et al., 2017).  **Ethics at the end of life** (n=5) (Ertugral et al., 2022; Kim et al..,2019; Qu et al., 2024; Yeom et al., 2017; Ziyai et al., 2024).  **Ethics at beginning of life** (n=4) (Kim et al., 2019; Yeom et al., 2017; Yuksel Kacan et al., 2022; Ziyai et al., 2024).  **Communication** (n=3) (Maddineshat et al., 201; Nesime et al., 2022; Zia et al., 2023).  **Dilemmas in mental health nursing** - (n-3) (Kim et al., 2019; Yeom et al., 2017; Yuksel Kacan, 2022).  **Drug admin/errors** (n=3) (Baykara et al., 2015; Ertugral et al., 2022; Ziyai et al., 2024).  **Patient rights** (n-3) (Baykara et al 2015, Kucukkelepce et al. 2020, Zia et al. 2023).  **Children’s issues** (n=2) (Yeom et al., 2017; Yuksel Kacan, 2022).  **Chronically ill** (n=2) (Yeom et al., 2017; Yuksel Kacan, 2022).  **Consent/refusal of treatment** (n=2) (Ertugral et al., 2022; Yeom et al., 2017).  **Inequalities in health** (n=2) (Nesime et al., 2022; Yeom et al., 2017).  **Interpersonal relationships** (n=2) (Kim et al., 2019; Qu et al., 2024).  **Legal issues** (n=2) (Baykara et al., 2015; Maddineshat et al., 2018).  **Medical technology** (n=2) (Qu et al., 2024 (telehealth), Yeom, 2017).  **Nursing research ethics** (n=2) (Baykara et al., 2015; Qu et al., 2024).  **Organ donation and Transplantation** (n=2) (Lee at al., 2017; Yeom et al., 2017).  **Bioethics** (n=1) (Maddenshatt et al., 2018).  **Confidentiality** (n=1) (Kim et al., 2019).  **Conflict between policy and nurses’ views** (n=1) (Kim et al., 2019).  **Advocacy** (n=1) (Nesime et al., 2022).  **Counselling skills** (n=1) (Nesime et al., 2022).  **Critical thinking** (n=1) (Yeom et al., 2017).  **Intersectionality/ discrimination** (Ageism) (n=1) (Yeom et al., 2017).  **New workforce** (n=1) (Ziyai et al., 2024).  **Nightingales’ diary** (n=1) (Lee et al., 2017).  **Nursing management** (n=1) (Qu et al., 2024).  **Patient safety in nursing care**  (n=1) (Ertugral et al., 2022).  **Public health services** (n=1) (Qu et al., 2024).  **Rest et al (1994) framework moral development** (n=1) (Morrill et al., 2022).  **Spirituality, religion, faith training** (n=1) (Ekramifar et al., 2018).  **Veracity** (n=1) (Ziyai et al., 2024).  35 codes  92 content suggestions | | **Case teaching and learning** (n=14) (Baykara et al., 2015; Ertugral et al., 2022; Ghoozlu et al., 2022; Kucukkelepce et al., 2020 ; Lee et al., 2017; Maddineshat et al., 2018; Morrill et al., 2022; Nesime et al., 2022; Park, 2011; Qu et al., 2024; Uncu et al., 2021; Yeom et al., 2017; Yuksel Kacal, 2022; Ziya et al. 2024).  **Lectures** (n=11) (Azarkish et al., 2023; Ekramifar et al., 2018; Jasemi et al., 2022; Kim et al., 2019; Kucukkelepce et al., 2020; Maddineshat et al., 2018; Morrill et al., 2022; Park, 2011; Park et al, 2012; Qu et al., 2024; Yeom et al. 2017).  **Discussions** (n=9) (Ertugral et al., 2022; Ekramifar et al., 2018; Lee et al., 2017; Morrill et al., 2022; Park, 2011; Park et al., 2012; Uncu et al., 202; Yeom et al., 2017; Yuksel Kacan, 2022).  **Visual material (video/movie)** (n=8) (Azarkish et al., 2023; Ertugral et al., 2022; Ghoozlu et al., 2023; Nesime et al., 2022; Qu et al., 2024; Yeom et al., 2017; Yuksel Kacal, 2022; Ziya et al., 2024).  **Slides** (n=7) (Azarkish et al., 2023; Ekramifar et al., 2018; Maddenshat et al., 2018; Morrill et al., 2022; Nesime et al., 2022; Qu et al., 2024; Zia et al. 2023).  **Question and answer methods**: (n=5) (Baykara et al., 2015; Ekramifar et al., 2018; Jasemi et al., 2022; Nesime et al., 2022; Yeom et al., 2017).  **Role play** (n=5) (Ertugral et al., 2022; Ghoozlu et al.,2023; Jasemi et al., 2022; Park, 2011; Yuksel Kacan, 2022).  **Reading material** (n=5) (Azarkish et al., 2023; Ekramifar et al,. 2018; Lee et al., 201; Yuksel Kacan, 2022; Ziyai et al., 2024).  **Scenarios** (n=4) (Ertugral et al., 2022; Jasemi et al., 2022; Lee et al., 2017; Zia et al., 2023).  **Simulation**(n=4) (Ghoozlu et al., 2023; Kucukkelepce et al., 2020; Qu et al., 2024; Sedgewick et al., 2019).  **Problem Based Learning** (n=4) (Azarkish et al., 2023; Park, 2011; Qu et al., 2024; Zia et al., 2023).  **Assignments** (n=3) (Lee et al., 2017; Maddineshat et al., 2018; Nesime et al., 2022).  **Debate** (n=2) (Kim et al., 2019; Maddineshat et al., 2018).  **Flipped learning** (n=2) (Azarkish et al., 2023; Ziyai et al., 2024).  **Mobile phone technology** (n=2) (Azarkish et al., 2023; Maddineshat et al., 2018).  **Projects** (n=2) (Ertugral et al., 2022; Lee et al., 2017).  **Reflection**(n= 2) (Morrill et al., 2022; Zia et al., 2023).  **Action plan** (n=1) (Nesime et al., 2022)  **Brainstorming** (n=1) (Lee et al., 2017).  **Concept Maps** (n=1) (Azarkish et al., 2023).  **Exam**(n=1) (Nesime et al., 2022)  **Field visits** (n=1) (Maddineshat et al., 2018).  **Games**(n=1) (Maddineshat et al., 2018).  **Jigsaw model** (n=1) (Ziyai et al., 2024).  **Literature searching** (n=1) (Lee et al., 2017).  **Observation** (n=1) (Nesime et al., 2022).  **Patient experiences** (n=1) (Lee at al., 2017).  **Poster presentation**(n=1) (Nesime et al.,2022)  **Quiz** (n=1) (Nesime et al., 2022)  **Role modelling**(n=1) (Morrill et al., 2022).  30 codes  102 activity suggestions | |
| **Constraints – Content.** | **Constraints – TALA** | **Moral decision making/judgement – Content.** | **Moral decision making/ judgement – TALA** |
|  |  | **Professional and ethical codes of nursing** (n=4) (Khatiban et al.,2019; Kim et al., 2019; Morrill et al .,2022; Park, 2011).  **Content to aid ethical decision making** (n=4) (Khatiban et al., 2019; Kim et al., 2019; Park, 2011; Ziyai et al., 2024).  **Ethical theories/frameworks/principles** (n=4)(Kim et al., 2019; Kucukkelepce et al., 2020; Park, 2011, Torabizadeh et al., 2016).  **Ethics at beginning of life** (n-=2) (Kim et al., 2019; Ziyai et al., 2024).  **Ethics for nursing practice** (n=2) (Park, 2011; Torabizadeh et al.,2016).  **Nursing Ethics** (n=2) (Kucukkelepce et al,. 2020; Park, 2011).  **Patients’ rights** (n=2) (Khatiban et al,. 2019; Kucukkelepce et al., 2020).  **Confidentiality** (n=1) (Kim et al., 2019).  **Conflict between policy and nurses’ views** (n=1)(Kim et al., 2019).  **Ethics at end of life** (n=1) (Kim et al., 2019).  **Ethics between coworkers/interpersonal relationships** (n=1) (Kim et al., 2019).  **Ethics in mental health nursing** (n=1)(Kim et al., 2019)  **New workforce** (n=1) (Ziyai et al., 2024).  **Rest et al (1994) framework moral development** (n=1) (Morrill et al., 2022).  **Veracity** (n=1) (Ziyai et al., 2024).  **Drug admin/error** (n=1) (Ziya et al., 2024).  16 codes  29 content suggestions | **Lectures** (n=7). (Khatiban et al., 2019; Kim et al., 2019; Kucukkelepce et al., 2020; Morrill et al., 2022; Park, 2011; Park et al., 2012; Torabizadeh et al., 2016).  **Case teaching and learning** (n=4) (Kucukkelepce et al., 2020; Morrill et al., 2022; Park, 2011; Ziyai et al., 2024).  **Discussion** (n=3) (Morrill et al., 2022; Park, 2011; Park et al., 2012).  **Problem based learning** (n=2) (Khatiban et al., 2019; Park, 2011).  **Reading material** (n=2) (Khatiban et al., 2019; Ziyai et al., 2024).  **Reflection** (n=2) (Morrill et al., 202; Torabizadeh et al., 2016).  **Scenarios** (n=2) (Khatiban et al., 2019; Torabizadeh et al.,2016).  **Debate** (n=1) (Kim et al., 2019).  **Flipped classroom** (n=1)(Ziyai et al., 2024).  **Jigsaw learning models** (n=1) (Ziyai et al., 2024).  **Role modelling** (n=1)(Morrill et al., 2022).  **Role play** (n=1) (Park, 2011)  **Simulation** (n=1) (Kucukkelepce et al., 2020).  **Slides/PowerPoint** (n=1) (Morrill et al., 2022).  **Socratic questioning/ question and answering** (n=1)(Torabizadeh et al., 2016).  **Visual material** (i.e videos) (n=1) (Ziyai et al., 2024).  16 codes  31 activity suggestions |
| **Moral courage – content** | **Moral courage - TALA** | **Moral resilience –Content.** | **Moral resilience – TALA** |
| **Ethics and law at end of life(**n=2) (Ford et al., 2024; Mattson, 2024).  **Content to aid ethical decision making** (n=2) (Ciesielski, 2022; Mattson, 2024).  **Ethics in nursing practice** (n=1) (Ciesielski, 2022).  **Conscientious objection** (n=1) (Ford et al., 2024).  **Disasters** (i.e climate change, pandemics). (n=1) (Ford et al., 2024).  **Patient safety, errors and reporting** (n=1) (Wawersick et al., 2023).  **Ethical theories** (n=1) (Mattson, 2024).  **Intersectionality/ discrimination** (n=1) (Ford et al., 2024).  **legal implications** (n=1) (Wawersick et al., 2023).  **Ethics and law in beginning of life** (n=1) (Ford et al., 2024).  **Implicit bias**(n=1) (Ford et al., 2024).  **Moral development** (n=1) (Mattson, 2024).  **Nursing skills** (n=1)(Mattson, 2024).  **Older person ethics** (n=1) (Mattson, 2024).  **Professional and ethical codes** (n=1) (Mattson, 2024).  **Scarce content** (n=1) (Ciesielski, 2022).  **TeamSSTEPS** (n=1) (Wawersick et al., 2023).  **Unethical practice** (n=1) (Ford et al., 2024).  18 codes  20 suggested topics | **Reflection** (n=3) (Ford et al., 2024; Mattson, 2024; Wawersick et al., 2023).  **Discussions**(n=3) - Teacher – student (Ciesielski, 2022),  Peer to peer discussions (Wawersick et al., 2023), online (Mattson, 2024).  **Learning environment** (n=2) (i.e safer environment, psychological safety, brave spaces, ground rules (Ford et al., 2024; Wawersick et al., 2023).  **Role Modelling (**n=1) (Wawersick et al., 2023).  **Assignments** (n=1) (Mattson, 2024).  **Case teaching and learning** (n=1) (Wawersick et al., 2023).  **Debriefing** (n=1) (Wawersick et al., 2023).  **Exam questions** (n=1) (Wawersick et al., 2023).  **Hidden curriculum activities** (n=1) (Ciesielski, 2022).  **Role play** (n=1) (Wawersick et al., 2023).  **Simulation (**n=1) (Wawersick et al., 2023).  **Mentoring/Coaching** (n=1) (Wawersick et al., 2023).  12 codes  17 activity suggestions | **Ethical frameworks, theories, principles** (n=3) **(**Forte et al.,2024; Monteverde, 2016; Rushton et al., 2017).  **Professional and ethical codes (**n=2) (Forte et al., 2024; Wros et al., 2021).  **Discussing moral resilience** (n=2) (Forte et al., 2024; Rushton et al., 2017).  **End of Life care ethics** (n=2) (Townsend et al., 2020; Wros et al., 2021).  **Mental health and Wellbeing** (n=2) (Townsend et al., 2020; Wros et al., 2021).  **Mental Health Nursing ethics**(n=2) (Kim et al., 2019; Monteverde, 2016).  **Work environment/ moral climate** (n=2) (Townsend et al., 2020; Wros et al.,( 2021).  **Advocacy** (n=1) (Wros et al., 2021).  **Beginning of life ethics** (n=1) (Townsend et al., 2020)  **Bioethics/Health care ethics**- not stated (n=1) (Wros et al., 2021).  **Compassion fatigue** (n=1) (Townsend et al., 2020).  **Constraints** (n=1) (Monteverde, 2016).  **Discussing moral distress** (n=1) (Townsend et al., 2020).  **Drug admin/ errors** (n=1) (Monteverde, 2016).  **Content to aid ethical reasoning/decision making** (n=1) (Monteverde, 2016).  **Ethical discourse** (n=1) (Wros. et al., 2021).  **I-Can programme** (n=1) (Wros et al., 2021).  **Incivility** (n=1) (Townsend et al., 2020).  **Interprofessional collaboration** (n=1) (Wros et al., 2021).  **Moving from Moral Distress to** **Moral Courage** (n=1) (Wros et al., 2021).  **Naming/framing / claiming Frameworks** (n=1) (Forte et al., 2024).  **Organ donation/ transplant** (n=1) (Wros et al., 2021).  **Organisational ethics and micro ethics** (n=1) (Wros et al., 2021).  **Personal and professional values** (n=1) (Wros et al., 2021).  **Sensory awareness skills** (n=1) (Forte et al., 2024).  **Social determinants of health** (external constraints) (n=1) (Wros et al., 2021).  26 codes  34 suggested topics | **Case teaching and learning** (n=2) (Monteverde, 201; Wros et al.,2021).  **Debriefing** (n=2) (Sedgewick et al., 2019; Wros et al., 2021**).**  **Simulation** (n=2) (Forte et al., 2024; Townsend et al., 2020).  **Lectures** (n=2) (Monteverde, 2016; Townsend et al., 2020).  **Assignment** (n=1) (Townsend et al., 2020).  **Coaching or mentorship (**n=1) (Wros et al., 2021).  **Didactic teaching** (n=1) (Forte et al., 2024)  **Discussions** (n=1) (Townsend et al., 2020).  **Ethics competencies students and faculty** (n=1) (Rushton et al., 2017).  **Learning environment** (safe spaces) (n=1) (Wros et al., 2021).  **Moral community development** (n=1) (Wros et al., 2021).  **Reading material** (n=1) (Townsend et al., 2020**).**  **Role modelling (instructor)** (n=1) (Wros et al., 2021).  **Role play** (n=1) (Townsend et al., 2020)  **Scenarios** (n=1) (Townsend et al., 2020).  **Slides/ Power point** (n=1) (Townsend et al., 2020).  **Visual material (i.e videos)** (n= 1) (Townsend et al., 2020).  17 codes.  21 Activity suggestions |
| **Moral Injury – Content.** | **Moral Injury – TALA** | **Moral Distress – Content.** | **Moral Distress – TALA** |
| **4 A's Model** (n=1) (Johansen et al., 2023**).**  **Conflict management** (n=1) (Johansen et al., 2023).  **Content to aid ethical decision making** (n=1) (Johansen et al., 2023).  **Disasters (i.e climate change, pandemics).** (n=1) (Johansen et al., 2023).  **Ethics content (not specified)** (n=1) (Johansen et al., 2023).  **Mental health/ wellbeing (post-traumatic stress)** (n=1) (Johansen et al., 2023).  6 codes.  6 topics. | **Case teaching and learning** (n=1) (Johansen et al., 2023).  **Debriefing (**n=1) (Johansen et al., 2023).  **Ethics webinar.** (n=1) (Johansen et al., 2023).  **Ethical competence** (n=1) (Johansen et al., 2023).  **Learning env** (**i.e Safe environment**) (n=1) (Johansen et al., 2023).  **Lecturing** (n=1) (Johansen et al., 2023).  **Role modelling** (n=1) (Johansen et al., 2023).  **Simulation** (n=1) (Johansen et al., 2023).  **Small groups** (n=1) (Johansen et al., 2023).  **Self-care assignment/ assignment** (n=1) (Johansen et al., 2023).  10 codes  10 activities | **Mental/Physical health and wellbeing strategies** (n=6)( Harvey et al., 2021; Jones-Schneck et al.,2021; Parker et al., 2024; Parsh et al., 2021; Wros et al., 2021; Yoes, 2012).  **Professional and ethical codes** (n=5) (Garity, 2009; Guzys, 2021; Parker et al., 2024; Robichaux et al., 2022; Wros et al., 2021).  **Content to aid ethical decision-making** (n=4) (Ciesielski, 2022; Garity, 2009; Harvey et al., 2021; Robichaux et al., 2022).  **4 A's Model** (n=3) (Parker et al., 2024; Parsh et al., 2021; Yoes, 2012).  **Advocacy** (n=3) (Guzys, 2021; Robichaux et al., 2022; Wros et al., 2021).  **Ethical theories, frameworks, principles** (n=3) (Garity, 2009; Guzys, 2021; Rushton et al., 2017).  **Conflict training/resolution** (n-=2) (Robichaux et al., 2022; Yoes, 2012).  **Ethics at the end of life** (n=2)(Garity, 2009; Wros et al., 2021).  **Ethics committees** (n=2) (Parsh et al., 2021; Robichaux et al., 2022).  **Scarce content** (n=2) (Ciesielski, 2022; Howarth, 2022).  **Work env or moral** **climate** (n=2) (Parker et al., 2024; Wros et al., 2021).  **Bioethics** (n=1) (Wros et al., 2021).  **Consent** **/ refusal of treatment**(n=1)(Harvey et al. (2021).  **Discussing moral distress** (n=1) (Howarth, 2022).  **Ethical discourse** (n=1) (Wros et al., 2021).  **Ethics at beginning of life** (n=1)(Garity, 2009).  **Ethics in nursing practice** (n=1) (Ciesielski, 2022).  **Emotional intelligence** (n=1) Robichaux et al., 2022).  **I-Can programme** (n=1) (Wros et al., 2021).  **Interprofessional collaboration** (n=1) (Wros et al., 2021).  **Organ donation/transplantation** (n=1) (Wros et al., 2021).  **Leadership** (n=1)(Guzys, 2021).  **Moral distress thermometer** (n-=1)(Parker et al., 2024).  **Moral distress toolkit** (n=1) (Jones – Schneck et al., 2021).  **Moving from Moral Distress to Moral Courage programme** (n=1)(Wros et al., 2021).  **Nursing ethics/philosophy** (n=1**)**  (Guzys, 2021).  **Organisational/Micro ethics** (n=1) (Wros et al., 2021).  **Professionals identify** (n=1) (Robichaux et al., 2022).  **Personal and professional values** (n=1) (Wros et al., 2021).  **RAISE programme** (n=1). (Parker et al., 2024).  **Social determinants of health** (n=1) (Wros et al., 2021).  **Self-efficacy.** (n=1) (Robichaux et al., 2022).  32 codes  55 topic suggestions | **Debriefs** (n=4)(Harvey et al., 2021; Parsh et al., 2021; Robichaux et al., 2022; Wros et al., 2021).  **Discussion** (n=4) Teacher – student (Ciesielski, 2022; Howarth, 2022; Jones – Schneck et al., 2021; Robichaux et al., 2022).  **Reflection** (n=4) (Ciesielski, 2022; Parker et al., 2024; Robichaux et al., 2022; Wros et al., 2021).  **Coaching/ Mentoring** (n=3) (Parker et al., 2024; Robichaux et al., 2022; Wros et al., 2021).  **Role modelling** (n=3)(Parker et al., 2024; Robichaux et al., 2022; Wros et al., 2020).  **Case teaching and learning** (n=3) (Garity, 2009: Howarth, 2022: Wros et al., 2021).  **Simulation** (n=2) (Harvey et al., 2021: Robichaux et al., 2022).  **Didactic teaching** – Type not specified (n=1) (Robichaux et al., 2022).  **Ethics competencies** (students and faculty) (n=1) (Rushton et al., 2017).  **Assignment writing** (n=1)(Garity, 2009).  **Debates** (n=1)(Garity, 2009).  **Ethics consultation** (n=1)(Parker et al., 2024).  **Journal clubs** (n=1)(Garity, 2009).  **Learning environment** (i.e safe spaces) (n=1)(Wros et al., 2021).  **Lecture** (n=1)(Harvey et al., 2021).  **Moral community development** (n=1) (Wros et al., 2021).  **Power point slides** (n=1)(Garity, 2009).  **Reading material** (n=1)(Garity, 2009).  **Role play** (n=1) (Robichaux et al., 2022).  **Visual material (i.e videos)** (n=1)(Garity, 2009).  20 codes.  36 activity suggestions |

***Once codes were established, a Tally table was created to count the overall number of cited content and TALA codes to avoid duplicate counting of the same code. Please contact the researchers should you have further questions.***
